# Candidate 3-benzazepine-1-ol type GluN2B receptor radioligands (^11^C-NR2B-Me enantiomers) have high binding in cerebellum but not to σ1 receptors

**DOI:** 10.1186/s13550-023-00975-6

**Published:** 2023-04-05

**Authors:** Lisheng Cai, Jeih-San Liow, Cheryl L. Morse, Sanjay Telu, Riley Davies, Lester S. Manly, Sami S. Zoghbi, Frederick T. Chin, Robert B. Innis, Victor W. Pike

**Affiliations:** 1 Molecular Imaging Branch, National Institute of Mental Health, National Institutes of Health, 10 Center Dr, Bldg 10, Room B3 C346, Bethesda, MD 20892 USA; 2 Molecular Imaging Program at Stanford, Department of Radiology, Stanford University, 1201 Welch Road, Rm. PS049, Stanford, CA 94305-584 USA

**Keywords:** GluN2B, NMDA receptor, σ1 receptor, NR2B-Me, PET

## Abstract

**Introduction:**

We recently reported ^11^C-NR2B-SMe ([*S*-*methyl*-^11^C](*R,S*)-7-thiomethoxy-3-(4-(4-methyl-phenyl)butyl)-2,3,4,5-tetrahydro-1*H*-benzo[*d*]azepin-1-ol) and its enantiomers as candidate radioligands for imaging the GluN2B subunit within rat *N*-methyl-D-aspartate receptors. However, these radioligands gave unexpectedly high and displaceable binding in rat cerebellum, possibly due to cross-reactivity with sigma-1 (σ1) receptors. This study investigated ^11^C-labeled enantiomers of a close analogue (7-methoxy-3-(4-(*p*-tolyl)butyl)-2,3,4,5-tetrahydro-1*H*-benzo[*d*]azepin-1-ol; NR2B-Me) of ^11^C-NR2B-SMe as new candidate GluN2B radioligands. PET was used to evaluate these radioligands in rats and to assess potential cross-reactivity to σ1 receptors.

**Methods:**

NR2B-Me was assayed for binding affinity and selectivity to GluN2B in vitro. ^11^C-NR2B-Me and its enantiomers were prepared by Pd-mediated treatment of boronic ester precursors with ^11^C-iodomethane. Brain PET scans were conducted after radioligand intravenous injection into rats. Various ligands for GluN2B receptors or σ1 receptors were administered at set doses in pre-blocking or displacement experiments to assess their impact on imaging data. ^18^F-FTC146 and enantiomers of ^11^C-NR2B-SMe were used for comparison. Radiometabolites from brain and plasma were measured ex vivo and in vitro.

**Results:**

NR2B-Me enantiomers showed high GluN2B affinity and selectivity in vitro. ^11^C-NR2B-Me enantiomers gave high early whole rat brain uptake of radioactivity, including high uptake in cerebellum, followed by slower decline. Radioactivity in brain at 30 min ex vivo was virtually all unchanged radioligand. Only less lipophilic radiometabolites appeared in plasma. When ^11^C-(*R*)-NR2B-Me was used, three high-affinity GluN2B ligands—NR2B-SMe, Ro25-6981, and CO101,244—showed increasing pre-block of whole brain radioactivity retention with increasing dose. Two σ1 receptor antagonists, FTC146 and BD1407, were ineffective pre-blocking agents. Together, these results strongly resemble those obtained with ^11^C-NR2B-SMe enantiomers, except that ^11^C-NR2B-Me enantiomers showed faster reversibility of binding. When ^18^F-FTC146 was used as a radioligand, FTC146 and BD1407 showed strong pre-blocking effects whereas GluN2B ligands showed only weak blocking effects.

**Conclusion:**

^11^C-NR2B-Me enantiomers showed specific binding to GluN2B receptors in rat brain in vivo. High unexpected specific binding in cerebellum was not due to σ1 receptors. Additional investigation is needed to identify the source of the high specific binding.

**Graphical Abstract:**

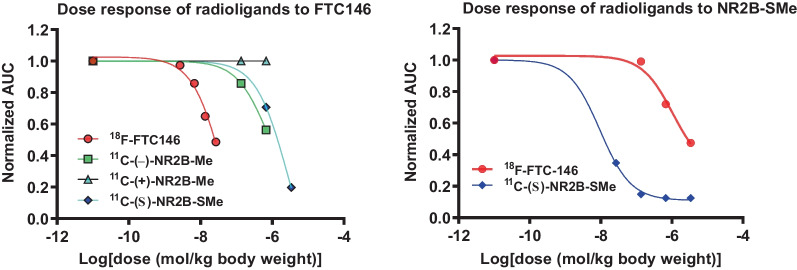

**Supplementary Information:**

The online version contains supplementary material available at 10.1186/s13550-023-00975-6.

## Key points

**Question**: Do GluN2B radioligands ^11^C-NR2B-Me enantiomers show cross-binding with σ1 receptors in brain in vivo?

**Pertinent findings**: ^11^C-(+)-NR2B-Me, ^11^C-(−)-NR2B-Me, ^11^C-(*S*)-NR2B-SMe, and ^11^C-(*R*)-NR2B-SMe give specific signals in whole rat brain that could be pre-blocked and displaced using ligands that specifically target GluN2B receptors, but not by σ1 ligand FTC-146.

**Implications for patient care**: ^11^C-(−)-NR2B-Me does not show cross-binding with σ1 receptor in rat brain using PET imaging. This radioligand may be promising for human PET imaging.

**Supplementary Information:**

The online version contains supplementary material available at 10.1186/s13550-023-00975-6.

## Introduction

*N*-Methyl-D-aspartate (NMDA) receptors are widely expressed throughout the central nervous system (CNS) and are involved in synaptic plasticity, learning, and memory. They are ligand- and voltage-gated ion channels that mediate the influx of Ca^2+^, Na^+^, and K^+^ into the synapse [1]. NMDA receptors exist as diverse tetrameric subtypes because they are assemblies of four subunits selected from seven different subunit types [GluN1, GluN2 (GluN2A − GluN2D), and GluN3 (A or B)]. Consequently, NMDA receptor subtypes have distinct physiological roles and pharmacological properties. In particular, NMDA receptors are implicated in major neuropsychiatric disorders, such as schizophrenia, pain, and clinical depression [2–5]. These receptors, especially those enriched with GluN2B subunits, endow the prefrontal cortex with important functionality as well as vulnerability to environmental insults and to risk factors for psychiatric disorders [6].

Sigma (σ) receptors are widely expressed in the CNS [7] and may function as a chaperone to NMDA receptors [8, 9]. They are involved in many normal physiological functions, such as neuronal firing, neurotransmitter release, learning, memory, and neuroprotection, and in pathological processes such as drug abuse [10]. Two subtypes exist, σ1 and σ2 [11]. Only the σ1 receptor has been cloned and extensively investigated. Upon activation by agonists, σ1 receptors translocate from the endoplasmic reticulum to the plasma membrane where they modulate both voltage-gated [12–16] and ligand-gated ion channels [17–19], including NMDA receptors [20]. σ1 receptors are widely distributed across the brain, including at low to medium levels in the cerebellum [21]. Many ligands that preferentially bind to the GluN2B subunit within NMDA complexes show cross-reactivity for σ receptors. For example, two well-known GluN2B receptor ligands, ifenprodil [22] and eliprodil [23], cross-react strongly with σ1 receptors, as do some 3-benzazepine-1-ols [24, 25].

**Fig. 1:**
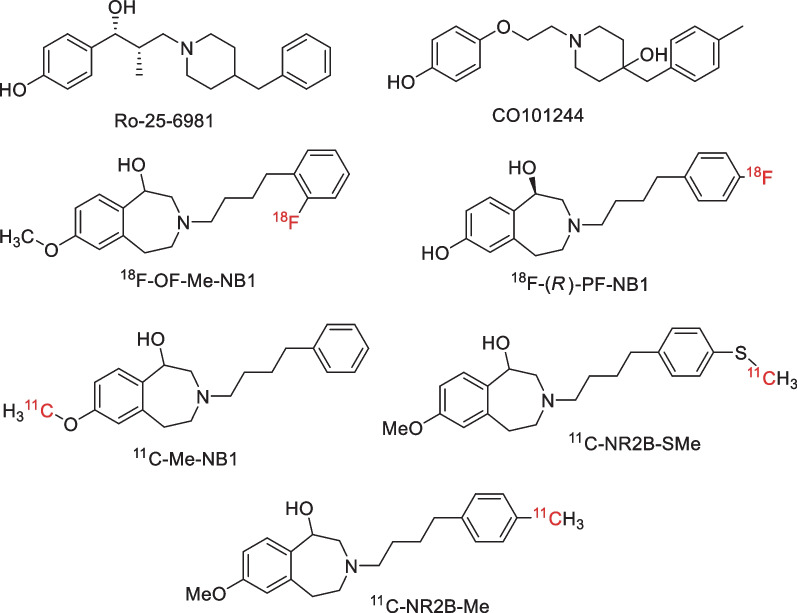
Structures of GluN2B ligands and candidate GluN2B radioligands

In a recent positron emission tomography (PET) study, we identified ^11^C-NR2B-SMe ([*S*-*methyl*-^11^C](*R,S*)-7-thiomethoxy-3-(4-(4-methyl-phenyl)butyl)-2,3,4,5-tetrahydro-1*H*-benzo[*d*]azepin-1-ol) and its enantiomers (Fig. 1) as candidate radioligands for imaging the GluN2B subunit of NMDA receptors within rat brain [26]. However, these 3-benzazepine-1-ol-type radioligands gave unexpectedly high and displaceable binding in rat cerebellum in vivo, suggesting that they might bind to an off-target site. Haider and colleagues [25] found that the (*R*)-enantiomer of the candidate 3-benzazepine-1-ol-type GluN2B radioligand ^18^F-OF-Me-NB1 (Fig. 1) was GluN2B receptor-preferring while the (*S*)-enantiomer was σ1 receptor-preferring in rodent brain in vitro. Although ^18^F-(*R*)-OF-Me-NB1 bound only a little to rodent cerebellum in vitro, PET imaging of rhesus monkeys found that this radioligand had only modest standardized uptake value ratios (SUVRs) relative to cerebellum of 1.37 (*P* = 0.001) for cortex, 1.30 (*P* = 0.002) for striatum, 1.36 (*P* = 0.003) for hippocampus, and 1.33 (*P* = 0.007) for thalamus.

In this study, the enantiomers of NR2B-Me—a close structural analog of NR2B-SMe—were labeled with carbon-11 (*t*_1/2_ = 20.4 min) as candidate high-affinity GluN2B radioligands (see Supplementary Information). The study sought to compare the binding of these new radioligands in rat brain with those of ^11^C-NR2B-SMe enantiomers and with ^18^F-FTC146, a known, highly selective σ1 receptor PET radioligand [27]. Fixed doses of ligands for GluN2B receptors and σ1 receptors were also used as pre-blocking or displacing agents to assess whether any radioligand showed cross-reactivity between GluN2B and σ1 receptors in vivo.

## Materials and methods

The Supporting Information provides all details on materials, general methods, statistics, measurement of absolute configuration, radiosyntheses, log*D* and p*Ka* measurement, radiometabolite analysis, and PET imaging in rats.

All experimental protocols were approved by the National Institute of Mental Health (NIMH) Animal Care and Use Committee. All methods were carried out in accordance with the *Guide for the Care and Use of Laboratory Animals* (https://grants.nih.gov/grants/olaw/guide-for-the-care-and-use-of-laboratory-animals.pdf). The study was carried out in compliance with the ARRIVE guidelines. No human subject is involved.

## Results

### Physical and pharmacological properties of NR2B-Me and its enantiomers

#### Absolute configuration

(−)-NR2B-Me and (+)-NR2B-Me were tentatively assigned *R* and *S* configuration, respectively, by comparing their order of elution in chiral HPLC with that of NR2B-SMe enantiomers of known absolute configuration [26] (Additional file 1: Figure S1). The labeling precursors, ‘(−)-NR2B Boron’ and ‘(+)-NR2B Boron’, were also tentatively assigned *R* and *S* configurations, respectively (Additional file 1: Figure S2).

#### Pharmacological screen

NR2B-Me at 10 μM concentration only weakly inhibited the binding of reference radioligands to numerous binding sites and receptors, as recoded in the Additional file 1. At this concentration, inhibition was greater than 10% for only a few binding sites and receptors: the calcium channel (39.6%), the hERG channel (61.7%), the guinea pig σ1 receptor (89.7%), and the PC12 cell σ2 receptors (90.7%).

#### Binding affinities in vitro

The *K*_i_ value for NR2B-Me measured in vitro in mouse fibroblast cells expressing NMDA was 4.9 nM (Table 1). (−)-NR2B-Me had higher affinity (*K*_i_, 42 nM) than its antipode (*K*_i_, 91 nM) for σ1 receptors. Both enantiomers showed *K*_i_ values of ≥ 100 nM for σ2 receptors.

**Table 1:** Physical and pharmacological parameters of FTC146, NR2B-SMe, NR2B-Me, and their enantiomers

Ligand	Ref	cLog*D*_7.4_	*p*K_a_	GluN2B *K*_i_ (nM)	σ1 *K*_i_ (nM)	σ2 inhibition^a^ (%)	σ2 *K*_i_ (nM)
FTC146	[27]	1.45	10.4		.0025		364
NR2B-SMe	[26]	3.41	5.03	2.2		90	
(*R*)-NR2B-SMe	[26]				88		110
(*S*)-NR2B-SMe	[26]				24		140
NR2B-Me		3.27	5.04	4.9		91	
(−)-NR2B-Me					42		110
(+)-NR2B-Me					130		100

^a^At 10 µmol

Table 1 also compares the physical and pharmacological properties of NR2B-Me enantiomers with those of the putative GluN2B ligands, NR2B-SMe, and its enantiomers [26] as well as with the σ1 receptor ligand FTC146 [27].

### Radiochemistry, and pKa and logD_7.4_ measurements

The precursors for the radiolabeling of ^11^C-NR2B-Me and its enantiomers were the corresponding boronic esters (Additional file 1: Figure S3). After reversed phase HPLC (see, for example, Additional file 1: Figure S4), each enantiomer of ^11^C-NR2B-Me was obtained ready for intravenous injection in 20 to 30% radiochemical yield from cyclotron-produced ^11^C-carbon dioxide and with molar activities of 58 to79 GBq/µmol in a radiosynthesis time of 40 min. Radiochemical purity was > 99% (Additional file 1: Figure S5). The p*K*a and log*D*_7.4_ of ^11^C-NR2B-Me were 5.03 and 3.07, respectively (Additional file 1: Figure S6).

### Experiments with ^11^C-NR2B-Me in rats and human brain homogenates and plasma

#### Stability of ^11^C-NR2B-Me in rat whole blood, plasma, and brain in vitro and ex vivo

Formulated ^11^C-NR2B-Me was at least 98.4% radiochemically stable at room temperature for the period encompassing tissue stability measurements (up to 3 h). ^11^C-NR2B-Me was 70.3% unchanged in rat plasma and completely unchanged in rat whole blood and brain homogenate at 37 °C after 30 min (Additional file 1: Table S1–S3).

At least 5 radiometabolites eluted before ^11^C-NR2B-Me in the reversed phase HPLC analyses of rat plasma ex vivo (Additional file 1: Figure S7A). These radiometabolites had very little presence in rat brain ex vivo (Additional file 1: Figure S7B). Unchanged radioligand at 30 min after injection accounted for 46.2% of radioactivity in rat plasma and 99.5% of radioactivity in rat brain (Additional file 1: Table S3). The brain showed high ratios of radioligand concentration to that in plasma (Additional file 1: Table S4). Radioactivity in plasma accounted for only a low percentage of radioactivity in blood, and most was bound with proteins in blood. From HPLC, the radiometabolites observed ex vivo (Additional file 1: Figure S7) appeared to match those seen in vitro*.*

#### Stability in human brain and plasma homogenate, and human plasma free fraction

^11^C-NR2B-Me was stable in human brain homogenate (99.5%) and human plasma (100%) at room temperature for at least 30 min. The human plasma free fraction (*f*_p_) of ^11^C-NR2B-Me was 1.16% ± 0.14% (*n* = 3).

### Evaluation of ^11^C-NR2B-Me enantiomers in rats using PET

Each ^11^C-NR2B-Me enantiomer gave similarly high and early peak radioactivity values in whole brain (~ 3.5 SUV within 3.5 min) after intravenous injection into rat (Additional file 1: Figure S8). The (−)-enantiomer (putative *R*-enantiomer) showed appreciably faster radioactivity decline from peak value than the (+)-enantiomer. In comparison, the enantiomers of ^11^C-NR2B-SMe showed very similar peak radioactivity uptake values but somewhat slower subsequent decline. Decline for the *R*-enantiomer was slightly faster than for the *S*-enantiomer (Additional file 1: Table S1-S2, Additional file 1: Figure S8).

**Fig. 2:**
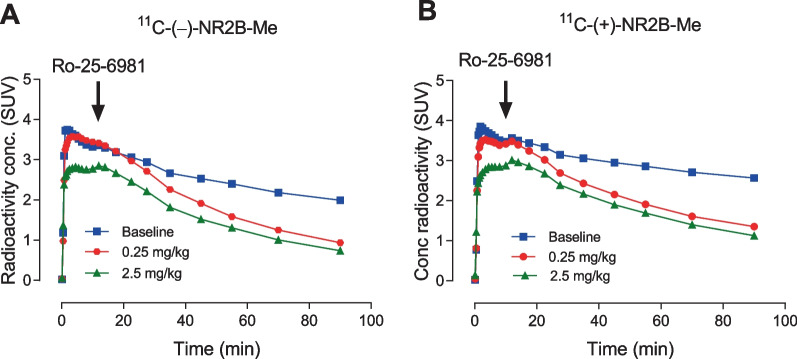
Whole brain time-activity curves for ^11^C-(−)-NR2B-Me (**A**) and ^11^C-(+)-NR2B-Me (**B**) with different displacement doses of the GluN2B ligand Ro-25-6981 at 10 min after radioligand injection. Displacement by 0.25 mg/kg of Ro-25-6981 at 90 min for panel **A** was 63%, whereas that for panel **B** was 46%. Data are for *n* = 1

Intravenous injection of the GluN2B ligand Ro-25-6981 at 10 min after each homochiral radioligand accelerated whole brain radioactivity washout (Fig. 2). The displacement of ^11^C-(−)-NR2B-Me was faster and more extensive than that of ^11^C-(+)-NR2B-Me. Thus, for a dose of 0.25 mg/kg of Ro-25-6981, displacement from peak value at 90 min reached 63% for ^11^C-(−)-NR2B-Me and 46% for ^11^C-(+)-NR2B-Me (Fig. 2).

**Fig. 3:**
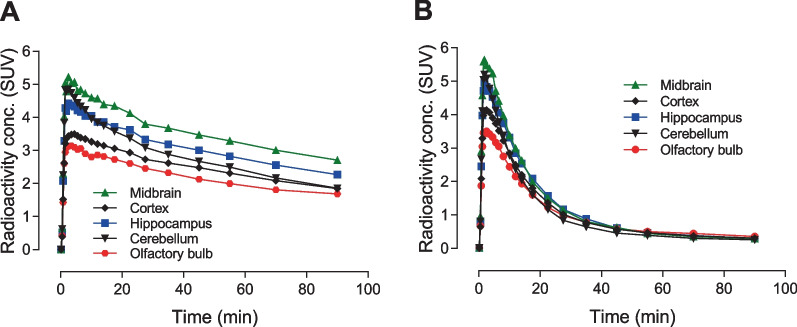
Time-activity curves for ^11^C-(−)-NR2B-Me in different regions of rat brain at baseline (**A**) and after pretreatment with Ro-25-6981 (0.25 mg/kg) (**B**). Data are for *n* = 1

A total of 14 regions were delineated on summed PET images of rat brain (0–90 min) (Additional file 1: Figure S9). Relatively high uptake was seen in the cortex and hippocampus. Lower levels were observed in the cerebellum, midbrain, and olfactory bulb (Fig. 3A). Intravenous administration of the GluN2B receptor ligand Ro-25-6981 (0.25 mg/kg) 10 min before ^11^C-NR2B-Me injection yielded peak uptake in brain regions, including cerebellum, that declined to a common level at 90 min, corresponding to about 10% of their peak values (Fig. 3B).

Lassen plots, using SUV as a surrogate for total binding of ^11^C-(−)-NR2B-Me or ^11^C-(+)-NR2B-Me in different regions of the rat brain at baseline and after pre-blocking with 0.25 mg/kg Ro-25-6981 gave slopes very close to unity, indicating near full receptor occupancy at this dose (Additional file 1: Figure S10). Non-displaceable binding given by the intercept on the X-axis of Lassen plots was, on average, about 0.42 SUV for each enantiomer. Estimates of *BP*_ND_ as (SUV_BL_/SUV_ND_−1) were 5.0 for the whole rat brain. *BP*_ND_ for ^11^C-(−)-NR2B-Me in different rat brain regions was between 3.4 and 7.6 and for ^11^C-(+)-NR2B-Me was between 4.6 and 6.9 (Additional file 1: Figure S10).

### Dose response of candidate GluN2B radioligands and ^18^F-FTC146 in whole rat brain to GluN2B pre-blocking agents

**Fig. 4:**
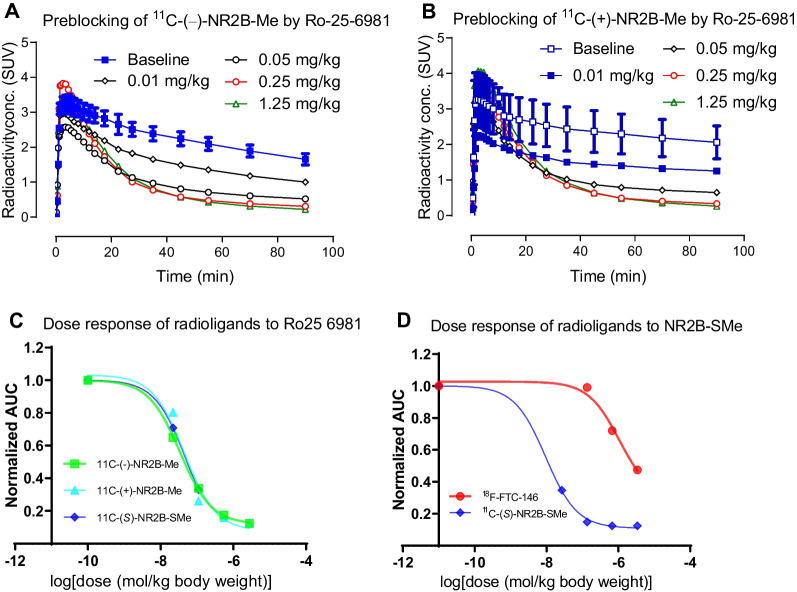
Blocking of whole brain radioactivity uptake in rat by dosing with the GluN2B ligand Ro-25-6981 before intravenous injection of ^11^C-(−)-NR2B-Me (**A**) or ^11^C-(+)-NR2B-Me (**B**), and the respective fitted dose–response curves of Ro-25 6981 from ^11^C-(−)-NR2B-Me, ^11^C-(+)-NR2B-Me, ^11^C-(*S*)-NR2B-SMe (*ED*_50_ = 34 nmol/kg, 45 nmol/kg, 29 nmol/kg) (**C**) and the respective fitted dose–response curves of Ro-25 6981 from ^18^F-FTC146, ^11^C-(*S*)-NR2B-SMe (*ED*_50_ = 1064 nmol/kg, 9.5 nmol/kg) (**D**). Data are for *n* = 1

AUCs (Area Under Curve) between 20 and 90 min for ^11^C-(−)-NR2B-Me and ^11^C-(+)-NR2B-Me, and between 20 and 120 min for ^18^F-FTC146—all administered with different doses of the GluN2B pre-blocking agent Ro-25-6981 (Fig. 4)—were used to measure *ED*_50_ values (Table 2). Similar experiments were performed with CO101,244 as the preblocking agent and ^11^C-(+)-NR2B-Me as the radioligand (Additional file 1: Figure S11). The *ED*_50_ values for Ro-25-6981 and CO101,244 versus the GluN2B radioligands in vivo generall*y* reflected their low *K*_i_ values measured in vitro. The *ED*_50_ values for (*S*)-NR2B-SMe versus ^18^F-FTC146 exceeded 1 µmol per kg body weight.

**Table 2:** In vitro and in vivo pharmacological parameters of GluN2B ligands

Ligand	*K*_i_ for GluN2B (nM)	*ED*_50_ in rat whole brain in vivo			
		^11^C-(S)-NR2B-SMe^a^	^11^C-(−)-NR2B-Me	^11^C-(+)-NR2B-Me	^18^F-FTC146
		(nmol/kg)			
NR2B-SMe	2.0	9.5			1064
Ro-25-6981	9.0	29	34	45	
CO101,244	43	74		41	

^a^Ref. [26]

^b^Ref. [36]

^c^Ref. [37]

### Dose response of candidate GluN2B radioligands and ^18^F-FTC146 in whole rat brain to σ1 receptor antagonists

**Fig. 5:**
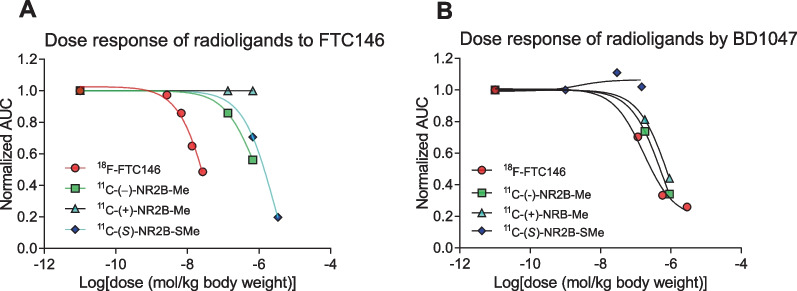
Rat whole brain uptake dose response for σ1 receptor radioligand ^18^F-FTC146 and GluN2B radioligands ^11^C-(−)-NR2B-Me, ^11^C-(+)-NR2B-Me, and ^11^C-(*S*)-NR2B-SMe to intravenous pre-administration of FTC146 (**A**) and BD1047 (**B**). Derived *ED*_50_ values are given in Table 3

Pre-administration of either of two σ1 receptor antagonists (FTC146 or BD1047) had minimal effects on whole rat brain radioactivity uptake for ^11^C-(*S*)-NR2B-SMe or the ^11^C-NR2B-Me enantiomers (Fig. 5; Table 3). *ED*_50_ values for the σ1 antagonists for blockade of ^18^F-FTC146 whole brain radioactivity uptake were far lower, in line with their low σ1 receptor *K*_i_ values measured in vitro*.*

**Table 3:** In vitro and in vivo pharmacological parameters for σ1 receptor ligands (not identified as agonists)

σ1 Ligand	*K*_i_ for σ1 (nM)^a^	*K*_i_ for σ2 (nM)^b^	*ED*_50_ in rat whole brain in vivo			
			^18^F-FTC146	^11^C-(−)-NR2B-SMe	^11^C-(+)-NR2B-Me	^11^C-(−)-NR2B-Me
			(nmol/kg)			
FTC146	0.0025^c,d^	364	46	2571	725	> 1000
BD1047	0.9^e^	47	169	> 1000	555	900

^a^Reference radioligand ^3^H-pentazocine, except that ^18^F-FTC146 was used for FTC146

^b^Reference radioligand ^3^H-DTG

^c^Ref. [27]

^d^Ref. [38]

^e^Ref. [39]

### Dose response of candidate GluN2B radioligands and ^18^F-FTC146 in whole rat brain to σ1 receptor agonists

The putative σ1 receptor agonists, TC1 and SA4503, showed strong pre-blocking effects on the whole rat brain uptake of the ^11^C-NR2B-Me enantiomers (Additional file 1: Figure S12 and Figure S13, respectively) (Table 4).

**Table 4:** In vitro and in vivo pharmacological parameters for putative σ1 receptor agonists

Ligand	*K*_i_ for σ1	Refs	*K*_i_ for σ2	*ED*_50_ in vivo versus^a^:	
				^11^C-(−)-NR2B-Me	^11^C-(+)-NR2B-Me
	(nM)^b^		(nM)^c^	(nmol/kg)	
TC1	10	[40]	370	48	64
SA4503	17.4	[41, 42]	1784	43	41
	4.6	[38, 43]	63		
(+)-Pentazocine	13.7	[41, 44]	2875	> 1000	
( ±)-PPCC	1.5	[45]	50.8	> 1000	
PRE-084	44	[46, 47]		> 1000	
(+)-SKF10047	48	[48]	625	> 1000	

^a^In rat whole brain

^b^Reference radioligand: ^3^H-pentazocine

^c^Reference radioligand: ^3^H-DTG

## Discussion

Although NR2B-Me was found to have high affinity for GluN2B, its affinity was nevertheless lower than earlier candidate GluN2B radioligands such as ^11^C-NR2B-SMe [26] or ^18^F-(*R*)-OF-Me-NB1 [25]. Each NR2B-Me enantiomer showed relatively much lower affinity for σ1 and σ2 receptors than for GluN2B receptors in vitro.

The lipophilicity of a PET radioligand, as indexed by log*D* at p*H* 7.4, is a key property that influences many aspects of PET radioligand behavior in vivo, including brain entry, metabolism, and protein binding [28]. Here, the log*D* of ^11^C-NR2B-Me was found to be 3.27, which is close to that predicted by computation (2.98) and in the range for many successful CNS PET radioligands. The plasma free fraction (*f*_p_) of a PET radioligand can be an important parameter for quantifying a receptor target in brain with compartmental models. *f*_p_ was low for ^11^C-NR2B-Me (1.16% ± 0.14%, *n* = 3) in human plasma but readily measurable with good precision. The apparent p*K*_*a*_ of ^11^C-NR2B-Me was 5.04 ± 0.01 (*n* = 3). Therefore free radioligand would be almost completely uncharged at physiological *p*H and available for brain entry.

^11^C-NR2B-Me was virtually unchanged when exposed to rat whole blood (Additional file 1: Table S3). Thus, blood samples could be analyzed without concern over further radioligand decomposition before measurement. ^11^C-NR2B-Me was also highly stable in brain homogenates (Additional file 1: Table S3). At 30 min post-intravenous administration, unchanged radioligand represented virtually all rat brain radioactivity (> 99%), a finding that was highly favorable to pursuing further radioligand characterization. Unchanged radioligand represented 46.2% of radioactivity in plasma at 30 min post-intravenous injection of ^11^C-NR2B-Me, showing that peripheral metabolism in vivo was relatively slow (Additional file 1: Table S3). ^11^C-NR2B-Me was stable in human brain homogenate (99.5%) and human plasma (100%) at room temperature for at least 30 min.

^11^C-(*-*)-NR2B-Me and ^11^C-(+)-NR2B-Me were compared with ^11^C-(*R*)-NR2B-SMe and ^11^C-(*S*)-NR2B-SMe in rat brain at baseline (Additional file 1: Figure S8). Each radioligand gave high and early whole brain radioactivity uptake that thereafter slowly declined. The rank order of radioactivity decline from peak was ^11^C-(−)-NR2B-Me > ^11^C-(+)-NR2B-Me > ^11^C-(*R*)-NR2B-SMe > ^11^C-(*S*)-NR2B-SMe. This order may reflect the lower GluN2B binding affinity of NR2B-Me than NR2B-SMe when measured in vitro. The density of GluN2B has been measured at 5.6 pmol/mg of protein in rat hippocampus [29], equivalent to 560 nM, which is a very high value compared to many PET imaging targets in brain [30]. This may be why moderately high-affinity GluN2B radioligands showed more evidence of reversible binding than very high affinity radioligands over the 90-min time course in our PET experiments.

To further explore how these new radioligands bind reversibly with GluN2B receptors, both pre-blocking and displacement of the PET signal with GluN2B ligands were examined. When the highly selective GluN2B ligand Ro-25-6981 (0.25 mg/kg) was intravenously injected 10 min before the radioligand, the PET signal in whole rat brain was reduced by up to 90% of that at baseline (Fig. 3). When Ro-25-6981 was injected 10 min after radioligand injection, radioactivity in whole brain declined smoothly and dose-dependently, although not to the same low level achieved in pre-blocking experiments by 90 min post-injection in PET imaging (Fig. 2). Corresponding experiments with ^11^C-NR2B-SMe had shown less extensive reversibility [26].

NR2B-SMe *ED*_50_ values for preblocking PET imaging signals from ^18^F-FTC146 and ^11^C-(*S*)-NR2B-SMe are 1064 nmol/kg and 9.5 nmol/kg, respectively (Table 2), indicating strong preference for binding of NR2B-SMe to the GluN2B site. FTC146 *ED*_50_ values for preblocking PET signals from ^18^F-FTC146 and ^11^C-(*S*)-NR2B-SMe are 46 nmol/kg and 2571 nmol/kg, respectively (Table 2), indicating strong preference for binding of FTC146 to the σ1 site (Table 3, Fig. 5A). The pre-blocking effect of FTC146 was weak against all four GluN2B radioligands (Fig. 5A). The σ1 receptor antagonist BD1047 was also less effective at blocking putative GluN2B radioligand uptake than the uptake of the σ1 receptor radioligand ^18^F-FTC146 (Fig. 5B). Like Ro-25-6981, the GluN2B ligand CO101,244 (Additional file 1: Figure S11) was also an effective pre-blocking antagonist against all four GluN2B radioligands. Collectively, these results provide strong evidence that the ^11^C-NR2B-Me enantiomers are selective for binding to GluN2B over σ1 receptors in rat brain. When we calculated *ED*_50_ values, we assumed that the preblocking agent distributed inside the rat body uniformly, as suggested by the unit of mg/kg, which is the measure of dose. We did not try to measure arterial input function in rat. We cannot say whether the preblocking agent had any effect on radioligand arterial input function or the plasma free fraction (*f*p) in rat. We assume that they did not change greatly. The high linearity and slope of the Lassen plots appear consistent with these assumptions. We used the flat part of the time-activity curves to construct the Lassen plots, because these likely represent a pseudo or near equilibrium state for the radioligand brain uptake. We believe our *ED*_50_ estimates are reasonably inter-comparable.

As previously observed for ^11^C-(*R*)-NR2B-SMe and ^11^C-(*S*)-NR2B-SMe [26], the putative σ1 receptor agonists TC1 and SA4503 showed strong pre-blocking effects on the whole rat brain uptakes of ^11^C-(−)-NR2B-Me and ^11^C-(+)-NR2B-Me (Additional file 1: Figure S12 and Figure S13, respectively). This supports our previous suggestion that TC1 and SA4503 interact directly with the GluN2B receptor, unlike the tested σ1 receptor antagonists [26].

Thalamus and cortex are generally considered to be GluN2B-rich regions. Here, we found that radioactivity retention in brain regions such as thalamus, cortex, and cerebellum could be pre-blocked with GluN2B ligands (Fig. 3). Both ^11^C-(−)-NR2B-Me and ^11^C-(+)-NR2B-Me showed high specific PET signal in rat brain (Fig. 3), with *BP*_ND_ reaching 5 in in rat whole brain, as assessed with Lassen (SUV) plots (Additional file 1: Figure S10).

Our finding that ^11^C-(−)-NR2B-Me gives substantial specific binding in cerebellum that can be blocked by Ro-25-6981 matches our previous findings with ^11^C-(*S*)-NR2B-SMe [26]. Together, they are consistent with the moderately high specific binding of the GluN2B radioligand (*R*)-^11^C-Me-NB1 seen in rat cerebellum in vivo [24]. Sixty minutes after injection of (*R*)-^11^C-Me-NB1, PET scanning revealed that radioactivity concentration in cerebellum was 79, 74, 75, and 83% of that in cortex, hippocampus, striatum, and thalamus, respectively. Ex vivo autoradiography of rat brain at 15 min after radioligand injection showed relatively lower binding in cerebellum than in, for example, cortex. (*R*)-^18^F-OF-Me-NB1 has also showed binding in rat cerebellum in vivo that could be blocked with eliprodil [25]. At 30 min after intravenous injection, radioactivity in cerebellum was 73, 74, 77, and 75% of that in cortex, hippocampus, striatum, and thalamus, respectively. A recent study also found that an isomerically related radioligand, (*R*)-^18^F-PF-NB1 (Fig. 1), bound to rat cerebellum in vivo, and that this binding could be pre-blocked with eliprodil [31]. At 45 min after intravenous injection, radioactivity in cerebellum was 85, 110, 95, and 92% of that in cortex, hippocampus, striatum, and thalamus, respectively. In summary, all tested candidate GluN2B radioligands from the 3-benzazepine-1-ol type structural class appear to show appreciable binding to cerebellum in vivo. In some cases, such as for the radioligands reported here, this uptake could be substantially blocked by recognized GluN2B ligands, such as Ro-25-6981.

In vitro autoradiography with ^3^H-Ro-25-6981 [29] and Western blot analysis using different antibodies against GluN2B [32–34] have been used to measure GluN2B protein levels in different regions of rat brain, including cerebellum. The mRNA of GluN2B has also been measured using hybridization histochemistry [1, 35]. Both protein and mRNA measurements suggest that the concentration of GluN2B receptors should be low in rat cerebellum in vivo. ^3^H-Ro-25-6981 showed weak binding to rat brain cerebellum in vitro*.* However, in this study we found that but Ro-25-6981 blocked ^11^C-NR2B enantiomer uptake in rat cerebellum in vivo at doses that were also effective in the rest of brain. Thus, it is possible that Ro-25-6981 is not wholly selective for GluN2B but also has high affinity for an unknown binding site. It is also possible that radioligands in the 3-benzazepine-1-ol class have strong affinity for a non GluN2B binding site.

## Conclusion

In this PET study, the new GluN2B radioligands ^11^C-(−)-NR2B-Me and ^11^C-(+)-NR2B-Me performed similarly to ^11^C-(*R*)-NR2B-SMe and ^11^C-(*S*)-NR2B-SMe but with faster washout from brain and more readily reversible specific binding to GluN2B receptors and with an absence of specific binding to σ1 receptors. However, the specific binding in cerebellum was unexpected from prior in vitro studies, and further investigation is warranted to unequivocally identify this binding. ^18^F-FTC146 is an established σ1 receptor PET radioligand, and this study further attests to its selectivity in vivo by showing a lack of cross reaction with GluN2B receptors.

### Supplementary information

The Supplementary Information file has information on: Materials: General methods; Chiral separations; Absolute configurations; Binding assay results; Radiosyntheses; Log*D* and pKa values; Radiometabolite analyses; Rat radioligand experiment results; Experiments with radioligands in human issue; Properties of NR2B-Me; Radiochemical stability; Additional file 1: Tables S1–S4; Additional file 1: Figures S1–S13.

## Supplementary Information

**Additional file 1**. Supplementary Methods, Tables and Figures.

## Data Availability

Not applicable.
