# Supplementary material for: Candidate 3-benzazepine-1-ol type GluN2B receptor radioligands (11C-NR2B-Me enantiomers) have high binding in cerebellum but not to σ1 receptors
Source: EJNMMI Res. 2023 Apr 5;13:28. doi: 10.1186/s13550-023-00975-6 (PMC10076467; doi:10.1186/s13550-023-00975-6)
Supplement: Supplementary file 1 — Additional file 1. Supplementary Methods, Tables and Figures. [file 13550_2023_975_MOESM1_ESM.docx]

*Cai et al — Candidate 3-benzazepine-1-ol type GluN2B receptor radioligands (^11^C-NR2B-Me enantiomers) have high binding in cerebellum but not to σ1 receptors*

**SUPPLEMENTAL INFORMATION**

**Supplemental Methods**

***Materials***

Reagents were purchased from Aldrich Chemical Co. (St. Louis, MI), Fluka Chemical Co. (Charlotte, NC), Acros (Waltham, MA), or Strem Chemicals ([Newburyport, MA](https://www.google.com/search?rlz=1C1GCEB_enUS849US849&sxsrf=ALiCzsbo3JCTvILQisOe7lKoYPrClCekfg:1662234098903&q=Newburyport&stick=H4sIAAAAAAAAAOPgE-LSz9U3MK5MSalKU-IAsUuqTAy1tLKTrfTzi9IT8zKrEksy8_NQOFYZqYkphaWJRSWpRcWLWLn9UsuTSosqC_KLSnawMu5iZ-JgAACy4crrWgAAAA&sa=X&ved=2ahUKEwi0lP7HsPn5AhWcGVkFHa6NB9wQmxMoAHoECEMQAg)). Solvents were obtained from Fisher Scientific (Waltham, MA). Water was purified with a Milli-Q system (Millipore, [Burlington, MA](https://www.google.com/search?rlz=1C1GCEB_enUS849US849&sxsrf=ALiCzsYf72BXVzUOReiz_fHJ-P5LjCnFwA:1662234216191&q=Burlington,+Massachusetts&stick=H4sIAAAAAAAAAOPgE-LSz9U3MKoyLjMtUuIEsQ1zjXILtbSyk63084vSE_MyqxJLMvPzUDhWGamJKYWliUUlqUXFi1glnUqLcjLz0kvy83QUfBOLixOTM0qLU0tKinewMu5iZ-JgAABbaeNdaQAAAA&sa=X&ved=2ahUKEwjT9PT_sPn5AhXnD1kFHTAkC6oQmxMoAHoECGoQAg)). Ligands used as pre-blocking or displacing agents, namely the GluN2B antagonists (Ro-25-6981 maleate and CO101,244 hydrochloride), the σ1 receptor agonists (TC1 and SA4503), and the σ1 receptor antagonist (BD1047) were purchased from Tocris (Minneapolis, MN).

Sprague Dawley rats were obtained from Taconic Farm (Germantown, NY) (188 male rats, about 4 to 12 weeks of age, and 100 to 250 g of weight). The usage of the rats is summarized in Supplementary Table S1). All animal experiments were performed in accordance with the *Guide for the Care and Use of Laboratory Animals* (<https://grants.nih.gov/grants/olaw/guide-for-the-care-and-use-of-laboratory-animals.pdf>) and were approved by the National Institute of Mental Health (NIMH) Animal Care and Use Committee.

***General Methods***

*Radioactivity Measurements*. γ-radioactivity from carbon-11 or fluorine-18 (>40 kBq) was measured with a calibrated dose calibrator (Atomlab 300; Biodex Medical Systems, Shirley, NY). Low radioactivity levels (<40 kBq) were measured with a calibrated automatic well-type γ‑counter (model 1480 Wizard; Perkin-Elmer, Waltham, MA) with an electronic window set between 360 and 1800 keV (counting efficiency, 51.8%). Carbon-11 radioactivity data were corrected for decay with a half-life of 20.4 minutes, and fluorine-18 radioactivity data were corrected for decay with a half-life of 109.8 minutes.

Radioactivity concentration in tissue was expressed as standardized uptake value (SUV), defined as (body weight /tissue weight) × (tissue activity/injected activity).

***Chiral Separations***

Samples of racemate solution were injected onto an (*S*,*S*)-Whelk-O1 column (21.1 × 250 mm; 5 µm; Regis Technologies Inc., Morton Grove, IL ) that was eluted at 80 mL/min with ethanol containing diethylamine (0.2% v/v) in liquid carbon dioxide (40:60 v/v) with eluate monitored for absorbance at 220 nm. Each enantiomer was isolated from the appropriate high-performance liquid chromatography (HPLC) fraction by evaporation of mobile phase under reduced pressure at 40 ^o^C. The purity of each enantiomer was determined via HPLC on an (*S*,*S*)-Whelk-O1 column (4.6 × 250 mm; 5 µm; Regis Technologies Inc.) eluted with hexane-0.2% diethylamine in ethanol (50:50 v/v) at 1.5 mL/min with eluate monitored for absorbance at 220 nm.

*Chiral Resolution of* *NR2B-Me.* NR2B-Me (1.1 g) was dissolved in ethanol (50 mL). Samples of this solution (1.5 mL) were injected onto the semi-preparative size chiral HPLC column. (+)-NR2B-Me (362 mg; *t*_R_ = 3.5 min) and (−)-NR2B-Me (351 mg; *t*_R_ = 5.3 min) were accumulated from these injections (Supplemental Figure S1). Each enantiomer was free of its antipode (i.e., had an *e.e.* of 100%).

*Chiral Resolution of* *NR2B-B(OR)_2_*. NR2B-B(OR)_2_ (0.128 g) was dissolved in ethanol (20 mL). Samples of this solution (1.0 mL) were loaded onto the semi-preparative size (*S,S*)-Whelk-O1 column to give **(+)-**NR2B-B(OR)_2_ (26 mg; *t*_R_ = 3.7 min) and (−)-NR2B-B(OR)_2_ (27 mg; *t*_R_ = 5.5 min) (Supplemental Figure S2). Each enantiomer was free of its antipode (i.e., had an *e.e.* of 100%).

***Tentative Assignment of Absolute Configurations***

The absolute configurations of (–)-NR2B-Me and (+)-NR2B-Me were tentatively assigned by comparing the order of their retention times in a chiral HPLC method with those of (–)-NR2B-SMe and (+)-NR2B-SMe for which the respective absolute configurations had been determined as *R* and *S* using vibrational circular dichroism (VCD) (*1*) (Supplemental Figure S1). Because this HPLC-type of assignment is not completely certain, the rest of this report continues to identify these enantiomers by the sign of their optical rotations, (+) or (‒). The absolute configurations of the enantiomers of the boronic acid precursors for labeling were also tentatively assigned with HPLC (Supplemental Figure S2).

***In Vitro Binding Assays***

NR2B-Me was assayed by the National Institutes of Health Psychoactive Drug Screening Program (PDSP; <http://pdsp.cwru.edu>) for 1/*K*_i_ at the NR2B subtype as an index of binding affinity. Thus, a suspension of transient transfected mouse fibroblast cell membrane homogenate was prepared as previously reported (*2*) at a concentration of ~500,000 cells per mL. This suspension was sonicated again and aliquots (100 μL) were added to each of four tubes (total 48 per rack). A solution of ^3^H-ifenprodil (2.22 TBq/mmol, 0.01 kBq/μL; Perkin–Elmer) in PBS (100 μL) was added to each tube. Non-radioactive ifenprodil or other displacer was dissolved in DMSO to give a 1.0 mM stock solution, which was further diluted with DMSO to give solutions ranging in known concentration from 10^-5^ to 10^-10^ M. Ten μL of each solution was then added to a separate tube. The content of each tube was then diluted to 1.0 mL with PBS, vortexed, and incubated at 37 °C for two hours. After separation of tube contents with a cell harvester, the filter paper (GF/B; Whatman), pretreated with 0.5% polyethyleneimine solution, was washed with PBS (3 mL × 3). Each filter was then placed in a 7-mL plastic vial. Scintillation fluid (4 mL) was added to each vial. The scintillation vials were incubated overnight and then counted for radioactivity. The data were analyzed with Prism 8 version 8.03 (GraphPad Software) with ‘One site competition’ curve-fitting. *K*_i_ values were calculated according to the Cheng-Prusoff equation (*3*): *K*_i_ = *IC*_50_/(1 + [L]/*K*_D_) where [L] is the concentration (0.4 nM) and *K*_D_ the equilibrium dissociation constant (7.6 nM) of the reference radioligand. The latter was determined with ‘Scatchard analysis’ of homologous displacement from multiple runs with self-displacement from membrane homogenates.

***Pharmacological Screen****.*

NR2B-Me was submitted to the PDSP for assessment of binding affinity against a wide range of other receptors and binding sites. A full listing of these receptors and binding sites is given in the Supplemental Results. Detailed assay protocols are available at the PDSP website (<http://pdsp.cwru.edu>).

***Radiosyntheses***

NR2B-Me, the ^11^C-NR2B-SMe enantiomers, and their precursors for radiolabeling were synthesized in house in high purity (>99% by HPLC) as previously described (*1*). ^18^F-FTC146 was also synthesized in house as previously described (*4*).

*Production of ^11^C-iodomethane*. No-carrier-added ^11^C-iodomethane was produced as previously described (*1*). Briefly, ^11^C-carbon dioxide (~ 50 GBq) was produced by irradiating nitrogen (initially ~225 psi) containing oxygen (1%) for 20 minutes with a proton beam (16 MeV, 40 µA) from a PETtrace cyclotron (GE Medical Systems). The ^11^C-carbon dioxide (>40 GBq) was converted into ^11^C-iodomethane by reduction to ^11^C-methane followed by high temperature iodination, all within a PETtrace Microlab module (GEMS PET Systems AB, Uppsala, Sweden).

*Preparation of ^11^C-NR2B-Me*. (±)-7-Methoxy-3-(4-(4-(4,4,5,5-tetramethyl-1,3,2-dioxaborolan-2-yl)phenyl)butyl)-2,3,4,5-tetrahydro-1*H*-benzo[d]azepin-1-ol (dubbed ‘NR2B-Boron’; 0.5 mg; 1.1 μmol) in methanol (0.40 mL) and cesium fluoride in methanol (5 μL of 1 M stock solution; 5 μmol) were loaded into a septum-sealed reaction vial (1-mL neck vial, Waters Corp., Milford, MA) that had been loaded with a mixture of Pd_2_(dba)_3_ and tri(2,4-dimethylphenyl)phosphine (1:4 mol ratio; 0.6 mg). ^11^C-iodomethane (>37 GBq) was then swept with a stream of helium (15 mL/min) into the vial from the Microlab module and heated at 80 ^o^C for five minutes (Supplemental Figure S3). ^11^C-NR2B-Me was separated out with HPLC on a Luna C18 column (10 μm, 10 × 250 mm; Phenomenex) eluted at 6 mL/min for 30 minutes with TFA (0.1% w/v) in methanol-water (50:50 v/v). Eluate was monitored for absorbance at 274 nm and for radioactivity (Supplemental Figure S4). The fraction containing ^11^C-NR2B-Me (*t*_R_ = 15.0 min) was collected in ascorbic acid solution (10 mg/mL; 0.1 mL) and then rotary evaporated to dryness (80 °C, water bath). The residue was dissolved in sterile saline for injection USP (4 mL) plus ethanol USP (1 mL) and aq. NaHCO_3_ solution (8.4% w/v; 40 µL). The solution (p*H* ~5.5) was sterile-filtered into a sterile and pyrogen-free dose vial that had been preloaded with sterile saline for injection USP (5 mL).

The formulated product was analyzed with HPLC on an X-Bridge C18 column (10 μm, 4.6 × 250 mm; Waters Corp.) eluted for 20 minutes with TFA (0.1% w/v) in methanol-water (50:50 v/v) at 1.5 mL/min (Supplemental Figure S5). Eluate was monitored for absorbance at 274 nm and for radioactivity. The retention time of ^11^C-NR2B-Me was 12 minutes. The response of the analytical system had been calibrated for mass of NR2B-Me to allow molar activity to be calculated.

The enantiomers of ^11^C-(-)NR2B-Me and ^11^C-(+)NR2B-Me were similarly prepared by using the appropriate homochiral NR2B-Boron precursor. Chiral HPLC analysis of residual carrier after radioactivity decay showed that no racemization had occurred during the radiosyntheses.

Each radioligand was identified with analytical HPLC by verifying its co-mobility with reference compound and with liquid chromatography-mass spectrometry (LC-MS) of residual carrier after full decay for comparison of its spectrum with that of reference compound. Formulated radioligand had greater than 99% radiochemical purity. The radiochemical stability of formulated racemic ^11^C-NR2B-Me at room temperature was assessed with radio-HPLC at three hours after radiosynthesis.

***LogD and pK_a_ Measurement***

For log*D* measurement (*5*), 1-octanol was pre-equilibrated overnight with sodium phosphate buffer (0.15 M; pH 7.4). ^11^C-NR2B-Me was dissolved in saline (0.9% w/v) containing ethanol (10% v/v) at a concentration of 159 MBq/mL. The radiochemical purity of the radioligand was measured via HPLC. Radioligand solution (~ 307 MBq; 900 µL) was added to sodium phosphate buffer (0.15 M; pH 7.4; 16.1 mL) and mixed well. Aliquots (1 mL) were distributed to each of eight borosilicate disposable culture tubes (13 × 100 mm). Six tubes were extracted with buffer pre-treated 1-octanol (1 mL) by vortexing for one minute and then centrifuged for one minute. The organic and aqueous phases were separated. Samples of organic phase (50 µL) and aqueous phase (50 µL) were taken and separately measured for radioactivity. The remaining two tubes containing radioactivity in buffer were analyzed with HPLC to check for any decrease in radiochemical purity over the time-span of the experiment. The radioactivity in the aqueous phase had a counting error of 0.4 ± 0.04% (*n* = 6) at one standard deviation. Each aqueous phase was analyzed via HPLC to determine radioligand purity (NR2B-Me, *t_R_* ~ 6.2 min) for use as a correction factor for the γ-counter counts. No correction for the γ-counting of the organic phase was needed because no radiochemical impurity was ever detected. Log*D* was calculated as log[(cpm/mL for 1-octanol phase)/(cpm/mL for aqueous phase)] using Pallas for Windows software (CompuDrug Inc.).

For p*K*_a_ measurement, the distribution of ^11^C-NR2B-Me between sodium phosphate buffers (0.15 M) of known p*H* and cyclohexane was measured following the methodology for log*D* determination (Supplemental Figure S6).

***Radiometabolite Analysis of ^11^C-NR2B-Me in Rat Tissues In Vitro***

Radiometabolites were analyzed via high performance liquid chromatography (HPLC) on an X-Terra C18 column (10 µm, 7.8 × 300 mm; Waters Corp.) housed within a compression module (Radial-Pak RCM-100; Waters Corp.) having a sentry pre-column. The column was eluted with MeOH:H_2_O:Et_3_N (82.5:17.5:0.1 by volume) at 4.0 mL/min. Eluted compounds were detected in series with an in-line photodiode-array absorbance detector (λ = 245 nm; Beckman Coulter, Sykesville, MD) with a flow-through Na(Tl) scintillation detector-rate-meter (Bioscan, Washington, DC). Samples were injected onto the HPLC column through nylon filters (13 mm × 0.45 µm; Iso-Disk; Supelco, Bellefonte, Pennsylvania). Recovery of all radioactivity from the HPLC column was checked by injection of absolute methanol (2 mL) at the end of chromatography with continued monitoring for radioactivity. Radiochromatograms were collected and stored with Bio-Chrome Lite software (Bioscan) and analyzed after decay correction.

*For Brain*. The brain (1.7 g) was homogenized in twice its volume of cold saline. Formulated ^11^C-NR2B-Me (20 µL; 3.7 MBq) was added to the homogenate, incubated at 37 °C for one hour, and then measured for radioactivity. An aliquot (450 µL) was then placed in acetonitrile (720 µL) along with carrier NR2B-Me (5 µg) and mixed well. Water (100 µL) was then added, mixed well, measured for radioactivity, and centrifuged at 10,000 *g* for one minute. The supernatant was analyzed via HPLC. The precipitate was measured for radioactivity to determine the recovery of radioactivity into the supernatant that had been injected onto HPLC.

*For Whole Blood*. Formulated ^11^C-NR2B-Me (10 µL; 1.85 MBq) was added to whole blood (200 µL). The sample was mixed well and incubated at 37 °C for one hour. Water (300 µL) was added to lyse open all cells. An aliquot (450 µL) was then removed to a new vial, and acetonitrile (720 µL) was added. The mixture was mixed well, measured for radioactivity, and centrifuged at 10,000 *g* for one minute. The supernatant was analyzed via HPLC as described for brain.

*For Plasma*. Plasma was separated from blood cells. Formulated ^11^C-NR2B-Me (10 µL; 1.85 MBq) was added to plasma (500 µL), mixed well, and incubated at 37 °C for one hour. The mixture was measured for radioactivity. An aliquot (450 µL) was then placed in acetonitrile (720 µL) along with carrier NR2B-Me (5 µg) and mixed well. Water (100 µL) was then added, mixed well, measured for radioactivity, and centrifuged at 10,000 *g* for one minute. The supernatant was analyzed with HPLC as described for brain. The precipitate was measured for radioactivity to determine radioactivity recovery into the supernatant that had been injected onto HPLC.

Finally, the metabolic stabilities of ^11^C-NR2B-Me in rat tissues *in vitro* were calculated by dividing the percentage of parent radioligand in the tissue sample measured with HPLC by the initial fractional radiochemical purity of the radioligand.

***Distribution of ^11^C-NR2B-Me in Rat Blood In Vitro***

Formulated ^11^C-NR2B-Me (1.85 MBq; ~ 10 µL) was added to anticoagulated rat whole blood (2.5 mL), mixed well, and incubated at 37 °C for 30 minutes. A sample of whole blood (50 µL) was measured for radioactivity. The remaining whole blood was centrifuged at 1,800 *g* for 2.5 minutes and an aliquot (50 µL) was removed for γ-counting. An aliquot (450 µL) of the plasma was then removed to a new vial. Acetonitrile (720 µL) was added, mixed well, and measured for radioactivity. The mixture was centrifuged at 10,000 *g* for one minute, and the supernatant was analyzed with via HPLC. The precipitate was measured for radioactivity to determine the recovery of radioactivity into the supernatant that was injected onto HPLC.

### The relative cellular blood (red blood cells, white blood cells, and platelets) partitioning of ^11^C-NR2B-Me was calculated with the following formula:

% *Cells* = [(*C*_whole blood_ – *C*_plasma_) × (1–Hcrt)]/*C*_whole blood_

where *C*_whole blood_ is the concentration of radioactivity in whole blood, *C*_plasma_ is the concentration of radioactivity in plasma, and *H*crt is the hematocrit. *H*crt was determined with a StatSpin® CritSpin™ Microhematocrit system (Beckman Coulter) using heparinized capillary tubes (12 × 40 mm) with blood samples drawn after the injection of the vehicle, immediately before injection of ^11^C-NR2B-Me.

**Experiments with ^11^C-NR2B-Me and Human Tissues *In Vitro***

*Metabolic Stabilities of ^11^C-NR2B-Me in Human Brain Homogenate and Human Plasma In Vitro.* The tissues had been stored frozen at −70 °C but were thawed for analysis. ^11^C-NR2B-Me (~37 kBq; 10.0 µL) was added to thawed tissue (650 µL), mixed well, and incubated for 30 minutes at room temperature. Subsequent processing and analysis were as described for rat *ex vivo* experiments in the main text.

*Human Plasma Free Fraction.* Plasma free fraction (*f*_p_) was measured as described previously (*6*). Briefly, formulated ^11^C-NR2B-Me (74 kBq; 11 µL) was mixed with human plasma (650 µL), incubated at room temperature for 10 minutes, and then filtered gravimetrically through ultrafiltration membranes (Centrifree; Millipore). If necessary, samples were left to decay to within the optimal radioactivity range for accurate γ-counter measurement.

***Radiometabolites of Racemic ^11^C-NR2B-Me in Rat Tissues Ex Vivo***

Rats were anesthetized with 1.5% isoflurane in oxygen. Formulated racemic ^11^C-NR2B-Me (200 µL; ~37 MBq) was then injected intravenously through the penile vein of each rat (see Supplemental Table S2 for additional experimental parameters). Thirty minutes after injection of ^11^C-NR2B-Me, a large anticoagulated (heparin) blood sample was drawn from each rat. The rats were then immediately sacrificed by decapitation, and their brains and myocardial tissues were excised.

For brain, the radioactive brain tissue was weighed, placed in acetonitrile (1 mL), measured for radioactivity, and homogenized along with carrier NR2B-Me (50 µg) using a hand-held tissue Tearor (model 985-370; BioSpec Products Inc., Bartlesville, OK). Water (500 µL) was then added, and the tissue was further homogenized before measuring radioactivity. The homogenate was centrifuged at 10,000 *g* for one minute. The supernatant was then analyzed with general HPLC as described above (NR2B-Me, *t_R_* ~ 6.4 min; Supplemental Figure S7), and the precipitate was measured for radioactivity to determine the recovery of radioactivity in the supernatant that had been injected onto the HPLC.

Plasma was separated from blood cells, and a sample (50 µL) was measured for radioactivity. An aliquot (450 µL) was placed in acetonitrile (720 µL) along with carrier NR2B-Me (5 µg) and mixed well. Water (100 µL) was added, mixed well, measured for radioactivity, and centrifuged at 10,000 *g* for one minute. The supernatant was analyzed with radio-HPLC and the precipitate measured for radioactivity, as described for brain.

***PET Imaging in Rats***

*Evaluation of Radioligands in Normal Rats with PET*

For radioligand injection, a polyethylene catheter (PE 10) was inserted into the penile vein of a male rat and then secured with tissue adhesive and tape. For dynamic scanning, the rats were secured with tape in the scanner and kept under 1.5% isoflurane anesthesia via a nose cone. Body temperature was monitored with a rectal probe. All PET scans were performed on a microPET Focus 220 scanner (Siemens Medical Solutions) (*7,8*). Data were acquired in listmode and reconstructed with Fourier rebinning + 2D filtered backprojection. A transmission scan was acquired before radioligand injection for attenuation correction. Dynamic scanning began at the time of a one-minute intravenous bolus injection of the radioligand under study (55 ± 19 MBq; volume 0.2 to 1.0 mL). The injected mass dose was 3 ± 1 nmol/kg. Challenge agents were administered intravenously 10 minutes before the radioligand for pre-blocking studies or 10 minutes after the radioligand for displacement studies. Tomographic images were analyzed with pixel-wise modeling computer software (PMOD 2.6; PMOD Group). Regions-of-interest in the brain were delineated, and time-activity curves calculated as SUV versus time.

*Estimation of ED_50_ Values In Vivo from Dose-response Data*

Pre-blocking and displacement experiments were performed to assess radioligand target engagement and radioligand selectivity *in vivo*. Normally 4 doses of 0.01 mg/kg, 0.05 mg/kg, 0.25 mg/kg, and 1.25 mg/kg were used for preblocking experiments, and only 2 doses of 0.25 mg/kg and 1.25 mg/kg were used for displacement experiments. These experiments used GluN2B receptor ligands (NR2B-SMe, Ro-25-6981, and CO101,244), σ1 receptor antagonists (FTC146 and BD1047), and σ1 receptor agonists (TC1 and SA4503) as challenge agents. After administering ^11^C-NR2B-Me or one of its enantiomers (^11^C-(*R*)-NR2B-SMe or ^18^F-FTC146) to rats at baseline, the time-course of whole brain radioactivity concentration was measured with PET and expressed as SUV. Areas-under the curve (AUCs) from time-activity curves between 20 and 90 minutes were calculated for each radioligand. AUCs obtained from the same production of radioligand were scaled to the value (or mean value) for the baseline experiment to give relative values (relative AUC_20–90 min_). These data were then used to estimate the dose of blocking agent that was effective for 50% reduction of the AUC_20–90 min_ at baseline (here termed *ED*_50_) and reported as nmol of administered blocker per kg of body weight. Where data permitted, the dose-response curves were fitted with GraphPad Prism software (version 8.1.1; GraphPad) to estimate the *ED*_50_ values. A dummy value of 100 pmol per kg body weight was used for zero concentration of the challenge agent in the construction of these dose-response curves. Numerical data are expressed as mean ± S.D. for *n*>2, or as mean and range for *n* = 2.

**Supplemental Results**

***Properties of NR2B-Me***

*Assessment of Binding Affinity against a Wide Range of Other Receptors and Binding Sites*. NR2B-Me was submitted to the National Institute of Mental Health Psychoactive Drug Screening program (NIMH-PDSP) to assess binding affinity against a wide range of other receptors and binding sites (5-HT_1A, 1B, 1D, 1E, 2A, 2A agonist, 2A antagonist, 2B, 2B agonist, 2B antagonist, 2C, 2C agonist, 2C antagonist, 3, 5A, 6, 7;_ α_2_; α2β2, α2β2, α3β2, α3β4, α4β2, α4β4, α7; α_1A, 1A agonist, 1B, 1B agonist, 1B antagonist, 2A-2C;_ AMPA*; β_1-3_; BZP_a1-3, a5, a6, rat brain site_; Ca^2+^-Channel; CB_1-2_, D_1, 2, 2 agonist, 2 antagonist, 3-5_; DAT; DOR; EP_1-4_; GABA_A,B_; GPR_40,41,43_; H_1-4_; HERG; IMIDAZOLINE 1; KA-R*; KOR, KOR_agonist, antagonist_; M_1, 1 agonist, 1 antagonist, 2, 3, 3 agonist, 3 antagonist, 4, 5, 5 agonist, 5 antagonist_; MDR 1; mGluR_1A,2,4,5,5-cloned,5-rat brain_,_6,8_; MOR; Na^+^-Channel; NET; NK_1, 1 antagonist, 2, 2 antagonist, 3, 3 antagonist_; NMDA; NMDA/MK801; NT-1; OXYTOCIN_agonist, antagonist_; PAR1_agonist, antagonist_; PCP; PKC_A, B, D, E, G_; PTHR2, Purinergic_agonist, antagonist_; SET; σ_1,2_; V_1, 1 agonist, 1 antagonist, 2, 2 agonist, 2 antagonist, 3, 3 agonist, 3 antagonist_, VMAT_1, 2_). At a concentration of 10 µM, inhibition was greater than 10% for only a few binding sites and receptors: calcium channel (39.6%), hERG channel (61.7%), guinea pig σ1 (89.7%), and PC12 cell σ2 (90.7%).

*Radiochemistry*. The radiosynthesis of ^11^C-NR2B-Me came from palladium-mediated methylation of the corresponding boronic ester (Supplemental Figure S3) with CsF. The ^11^C-iodomethane was synthesized according to a method previously established in our laboratory. After reversed phase HPLC, each enantiomer of ^11^C-NR2B-Me was obtained ready for intravenous injection in 20 to 30% radiochemical yield from cyclotron-produced ^11^C-carbon dioxide and with molar activities of 58 to 79 GBq/µmol in a radiosynthesis time of 40 minutes. Radiochemical purity was >99% (Supplemental Figure S5).

*LogD and pK_a_ of ^11^C-NR2B-Me*. The measured log*D*_7.4_ of ^11^C-NR2B-Me at room temperature in 1-octanol was 3.27 ± 0.08 (*n* = 6) and in fair agreement with the value (clog*D*_7.4_) of 2.98 computed with Pallas software. The apparent p*K*_a_ was found to be 5.04 ± 0.27 (*n* = 3) (Supplemental Figure 6).

**Stability of ^11^C-NR2B-Me in the Formulated Dose**

The radiochemical stability of ^11^C-NR2B-Me was found to depend on the composition of the formulation. The radiochemical purity of ^11^C-NR2B-Me ranged between 95 and 99% immediately after radiosynthesis. However, the fraction collected from HPLC contains 0.1% TFA. When the p*H* of this fraction was not increased immediately before drying by rotary evaporation at 80 ^o^C, the purity measured for the formulated radioligand was only about 90%. Protection with ascorbic acid was therefore used immediately after HPLC radioligand purification to maintain weakly acidic and reducing conditions. The formulated radioligand was then radiochemically stable for at least three hours at room temperature (99.9 ± 0.3%; *n* = 4), and stable in PBS (10 mM) for at least 2.5 hours. There was virtually no adsorption of radioligand to the walls of glass test tubes when in PBS (10 mM) (98.1 ± 0.5%; *n* = 4, the radioligand remained in solution). The error in the γ-counter measurements of samples with least counts was 0.3 ± 0.12% (*n* = 6).

**Experiments with ^11^C-NR2B-Me in Rats and Human Tissues**

*Stability of ^11^C-NR2B-Me in Rat Whole Blood, Plasma, and Brain Ex Vivo and In Vitro*. ^11^C-NR2B-Me was slightly unstable in rat plasma *in vitro*, with about 30% of a single less lipophilic radiometabolite **(***t_R_*~2.8 min) detected after incubation for 30 minutes at 37 °C. ^11^C-NR2B-Me was especially stable in brain homogenate, with less than 0.5% conversion into radiometabolite. The stability of ^11^C-NR2B-Me in rat tissues *ex vivo* was obtained by dividing the percentage of radioactivity present as radioligand in the tissue sample measured with HPLC by the fractional radiochemical purity of the radioligand (Supplementary Table S3). The SUV due to radioligand or radiometabolite only was calculated by multiplying the total SUV of the tissue by the fraction of the radioligand or radiometabolite measured with HPLC (Supplementary Table S4).

*Metabolic Stability of ^11^C-NR2B-Me* *in Human Brain Homogenate and Human Plasma*. ^11^C-NR2B-Me was stable in human brain homogenate (99.5%) and human plasma (100%) at room temperature for at least 30 minutes.

**Supplemental Tables**

**Supplementary Table S1**. The number of rats assigned for each radioligand. A total of 188 rats were used in the in vitro, ex vivo, PET imaging baseline, PET imaging preblocking, and PET imaging displacing experiments (see *Materials* for details).

| # | **Radioligand** | **Purpose** | **Rats used** |  | **#** | **Radioligand** | **Purpose** | **Rats used** |
| --- | --- | --- | --- | --- | --- | --- | --- | --- |
| 1 | ^11^C-(–)-NR2B-Me | In vitro | 1 |  | 2 | ^11^C-(–)-NR2B-Me | Ex vivo | 3 |
| 3 | ^11^C-(–)-NR2B-Me | PET baseline | 5 |  | 4 | ^11^C-(+)-NR2B-Me | PET baseline | 5 |
| 5 | ^11^C-(*R*)-NR2B-SMe | PET baseline | 1 |  | 6 | ^11^C-(*S*)-NR2B-SMe | PET baseline | 5 |
| 7 | ^11^C-(*R*)-NR2B-SMe | Preblocker Ro256981 | 6 |  | 8 | ^11^C-(*S*)-NR2B-SMe | Preblocker Ro256981 | 6 |
|  |  |  |  |  | 9 | ^11^C-(+)-NR2B-Me | Preblocker Co101244 | 6 |
| 10 | ^11^C-(–)-NR2B-Me | Preblocker TC1 | 6 |  | 11 | ^11^C-(+)-NR2B-Me | Preblocker TC1 | 6 |
| 12 | ^11^C-(–)-NR2B-Me | Preblocker SA4503 | 6 |  | 13 | ^11^C-(+)-NR2B-Me | Preblocker SA4503 | 6 |
| 14 | ^11^C-(–)-NR2B-Me | Displacer Ro256981 | 3 |  | 15 | ^11^C-(+)-NR2B-Me | Displacer Ro256981 | 3 |
| 16 | ^11^C-(–)-NR2B-Me | Preblocker Ro256981 | 6 |  | 17 | ^11^C-(+)-NR2B-Me | Displacer Ro256981 | 6 |
| 18 | ^11^C-(–)-NR2B-Me | Preblocker FTC146 | 6 |  | 19 | ^11^C-(+)-NR2B-Me | Displacer FTC146 | 6 |
| 20 | ^11^C-(–)-NR2B-Me | Preblocker BD1047 | 6 |  | 21 | ^11^C-(+)-NR2B-Me | Displacer BD1047 | 6 |
| 22 | ^11^C-(*S*)-NR2B-SMe | Preblocker FTC146 | 6 |  | 23 | ^11^C-(*S*)-NR2B-SMe | Preblocker BD1047 | 6 |
| 24 | ^18^F-FTC146 | Preblocker FTC146 | 6 |  | 25 | ^18^F-FTC146 | Preblocker BD1047 | 6 |
| 26 | ^18^F-FTC146 | Preblocker NR2B-SMe | 6 |  | 27 | ^11^C-(*S*)-NR2B-SMe | Preblocker Co101244 | 6 |
| 28 | ^11^C-(–)-NR2B-Me | Preblocker Pentazocine | 6 |  | 29 | ^11^C-(+)-NR2B-Me | Displacer Pentazocine | 6 |
| 30 | ^11^C-(–)-NR2B-Me | Preblocker  (±)PPCC | 6 |  | 31 | ^11^C-(+)-NR2B-Me | Displacer (±)PPCC | 6 |
| 32 | ^11^C-(–)-NR2B-Me | Preblocker  PRE-084 | 6 |  | 33 | ^11^C-(+)-NR2B-Me | Displacer PRE-084 | 6 |
| 34 | ^11^C-(–)-NR2B-Me | Preblocker  (+)-SKF10047 | 6 |  | 35 | ^11^C-(+)-NR2B-Me | Displacer  (+)-SKF10047 | 6 |

**-**

**Supplemental Table S2**. Experimental parameters used for the analysis of ^11^C-NR2B-Me radiometabolites in whole blood, plasma, and brain of one rat *in vitro* at 37 °C and three rats *ex vivo*, and recoveries of radioactivity into MeCN from tissues (see *Radiometabolites of Racemic ^11^C-NR2B-Me in Rat Tissues Ex Vivo* for details).

| **Sample** | ***In vitro*** | ***Ex vivo*** |
| --- | --- | --- |
| Injected radioactivity (MBq) |  | 898±350 |
| Rat weight (g) | 441 | 362±33 |
| Molar activity at injection time (MBq/µmol) |  | 1107±219 |
| Mass injected (nmol/kg) |  | 2.33±1.01 |
| Experiment duration (min) | 30 | 30 |
| **Recoveries (%) into MeCN from (%):** | | |
| Plasma | 94.8 | 92.5±0.4 |
| Whole blood | 95.5 |  |
| Brain | 94.1 | 84.6±0.5 |

**Supplemental Table S3**. *Ex vivo* and *in vitro* stabilities of ^11^C-NR2B-Me in rat tissues. Measurements were made 30 minutes after intravenous injection of radioligand *ex vivo* and 30 minutes after incubation of radioligand at 37 °C *in vitro*. Stabilities are represented by the ratios of the percentage of the parent radioligand in the tissue to the fractional radioligand purity at the start of the study (see *Experiments with ^11^C-NR2B-Me in Rats and Human Tissues* for details).

| **Radioactive sample*^a^*** | ***In vitro* stability**  **(%)** | ***Ex vivo* stability**  **(%)** |
| --- | --- | --- |
| Whole blood | 98.1 |  |
| Plasma | 70.3 | 46.2 |
| Brain | 98.7 | 99.5 |

*^a^* The formulated radioligand was initially 98.4% pure and found to be 99.2% intact 3 hours later, when other measurements had concluded.

**Supplemental Table S4**. Concentrations of ^11^C-NR2B-Me in rat tissues, and tissue to plasma radioactivity ratios measured *ex vivo* 30 minutes after intravenous injection (see *Experiments with ^11^C-NR2B-Me in Rats and Human Tissues* for details).

| **Radioactive sample** | **Radioactivity conc.**  **(SUV)** | **Tissue to plasma radioactivity ratio** |
| --- | --- | --- |
| Whole blood | 0.300 | 7.5 |
| Plasma | 0.04 | 1.0 |
| Brain | 4.1 | 103 |

1. (*+*)-NR2B-Me
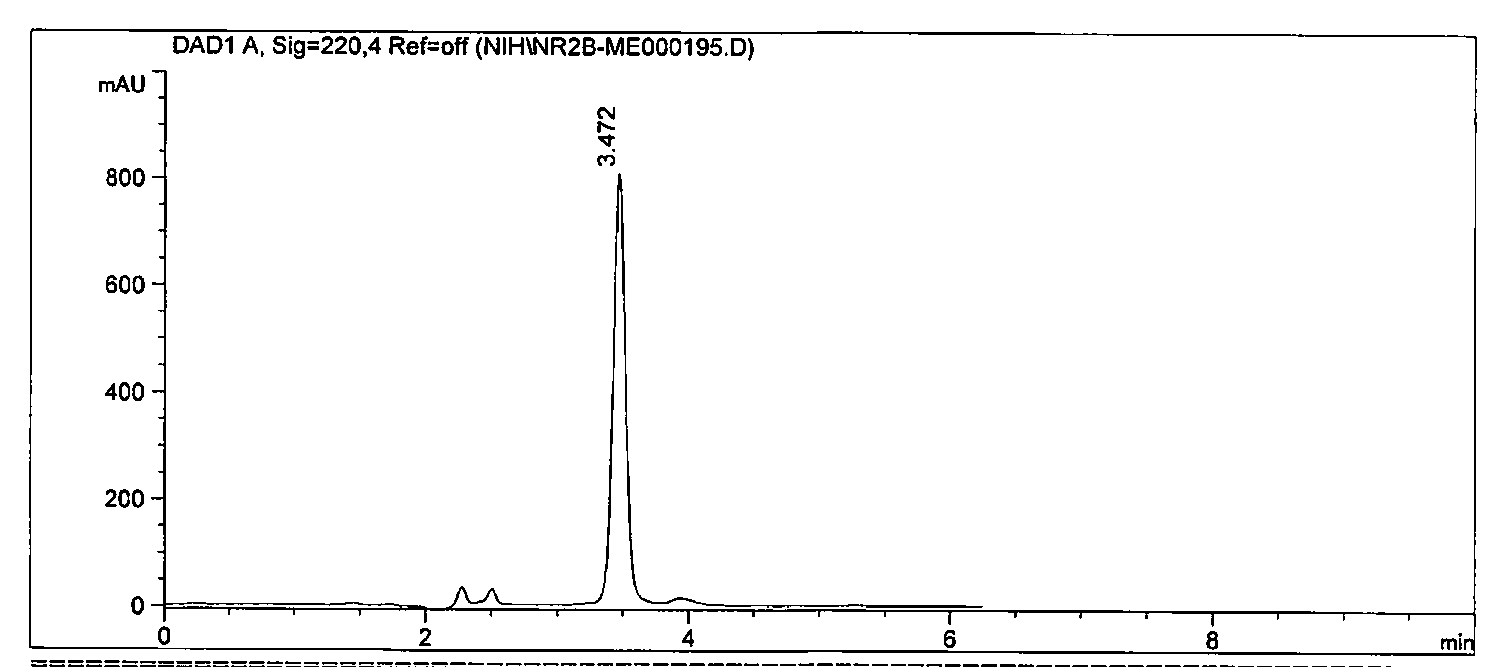

2. (*–*)-NR2B-Me
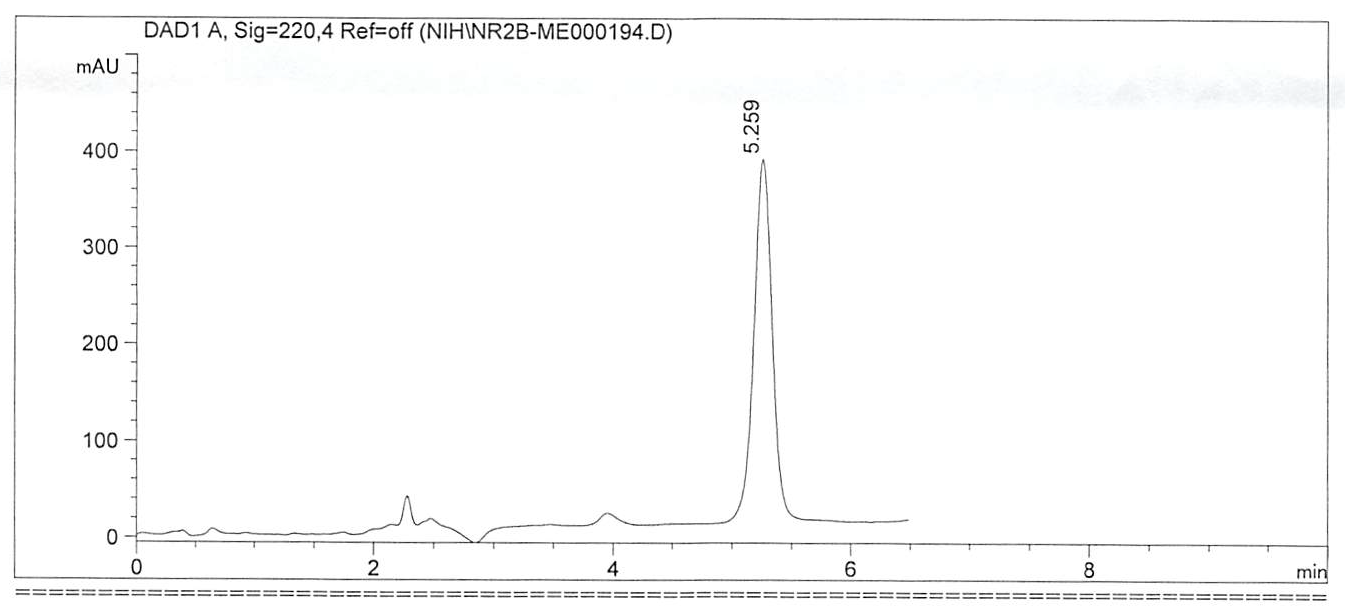


Time (min)

**Supplemental Figure S1**. Chiral high-performance liquid chromatography (HPLC) of the enantiomers of NR2B-Me. *HPLC conditions*: (*S,S*)-Whelk-01 column (250 × 4.6 mm; 5 µm) eluted with hexane/ethanol (1:1 v/v) containing 0.1% diethylamine at 1.5 mL/min. Injection volume 50 µL; *t*_R_: (*+*)-NR2B-Me, 3.47 min; (–)-NR2B-Me, 5.26 min (see *Chiral Separations* for details).

1. (*+*)-NR2B-B(OR)_2_


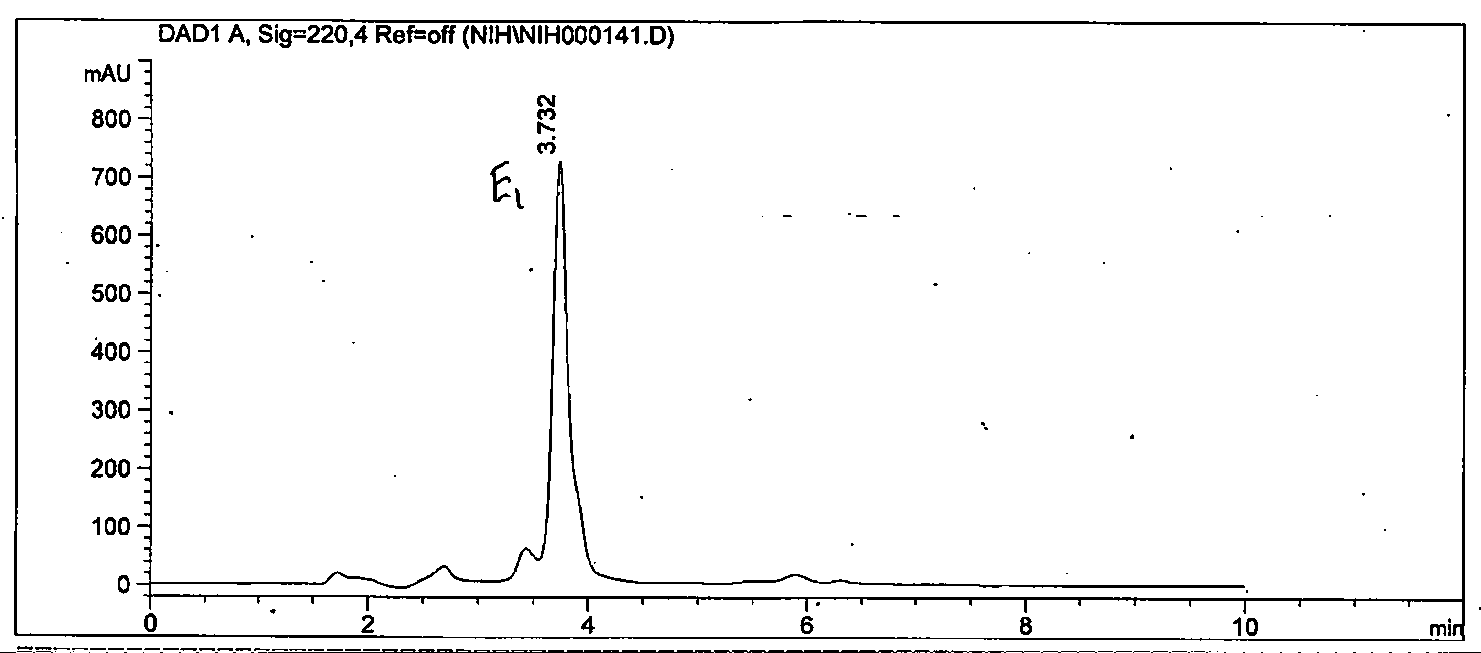


1. (*–*)-NR2B-B(OR)_2_


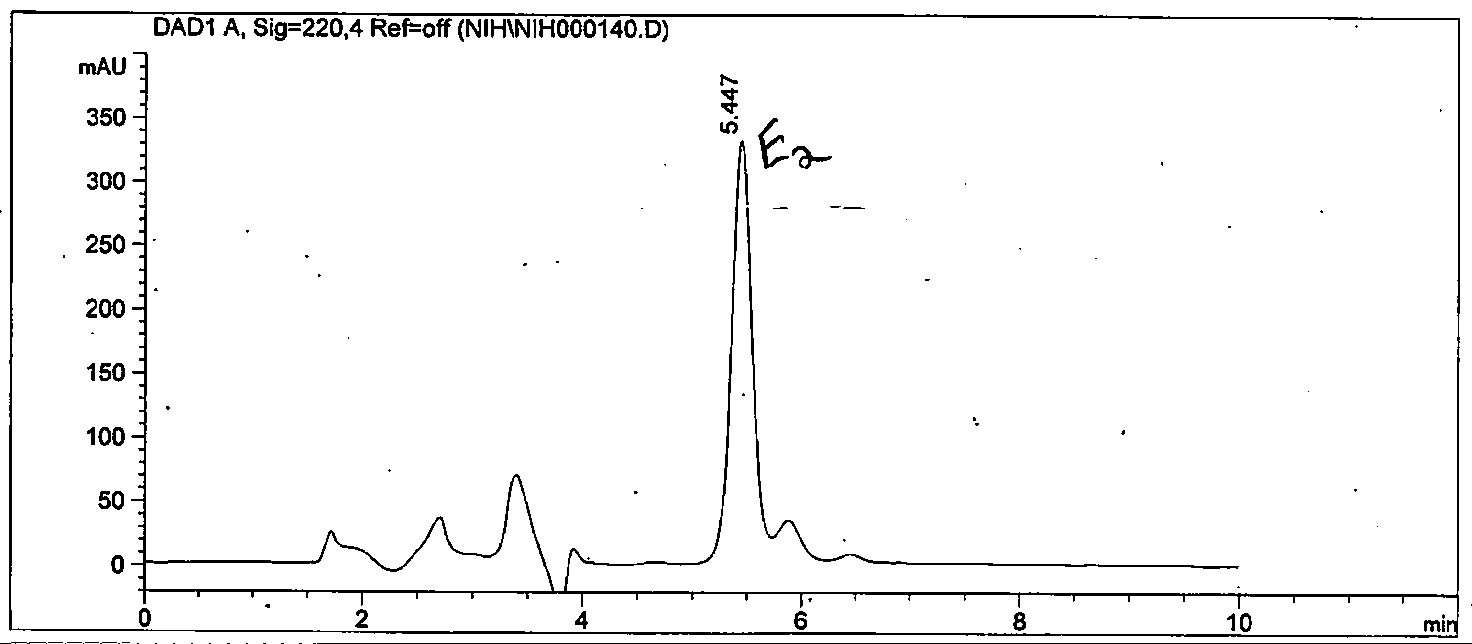


Time (min)

**Supplemental Figure S2**. Chiral high-performance liquid chromatography (HPLC) of the enantiomers of NR2B-B(OR)_2_. *HPLC conditions*: (*S,S*)-Whelk-O1 column (250 × 4.6 mm; 5 µm) eluted with hexane/ethanol (1:1 v/v) containing 0.1% diethylamine at 1.5 mL/min. Injection volume 50 µL; *t*_R_: (*S*)-NR2B-B(OR)_2_, 3.72 min; (*R*)-NR2B-B(OR)_2_, 5.45 min. (see *Chiral Separations and Absolute Configurations* for details).

**Supplemental Figure S3**. Radiosynthesis of ^11^C-NR2B-Me from boronic acid precursor (‘NHR2B boron’) and ^11^C-methyl iodide. The enantiomers were prepared from the corresponding homochiral precursors (see *Properties of NR2B-Me* for details).


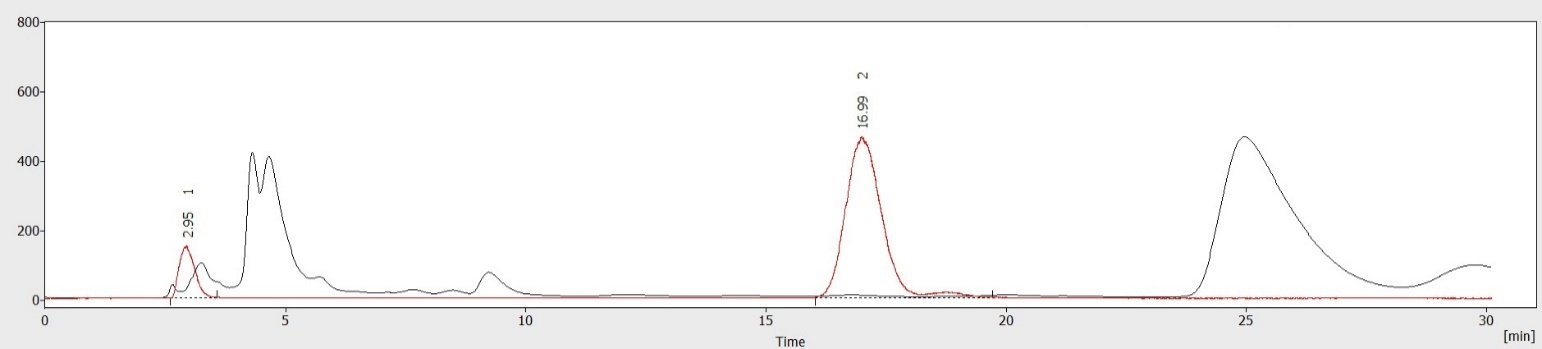


**Supplemental Figure S4.** Radiochromatogram from the high-performance liquid chromatography (HPLC) separation of ^11^C-(+)-NR2B-Me after radiolabeling. Y-axes are on linear scales. Black line: absorbance at 274 nm. Red line: radioactivity detector response (see *Radiosyntheses* for details).


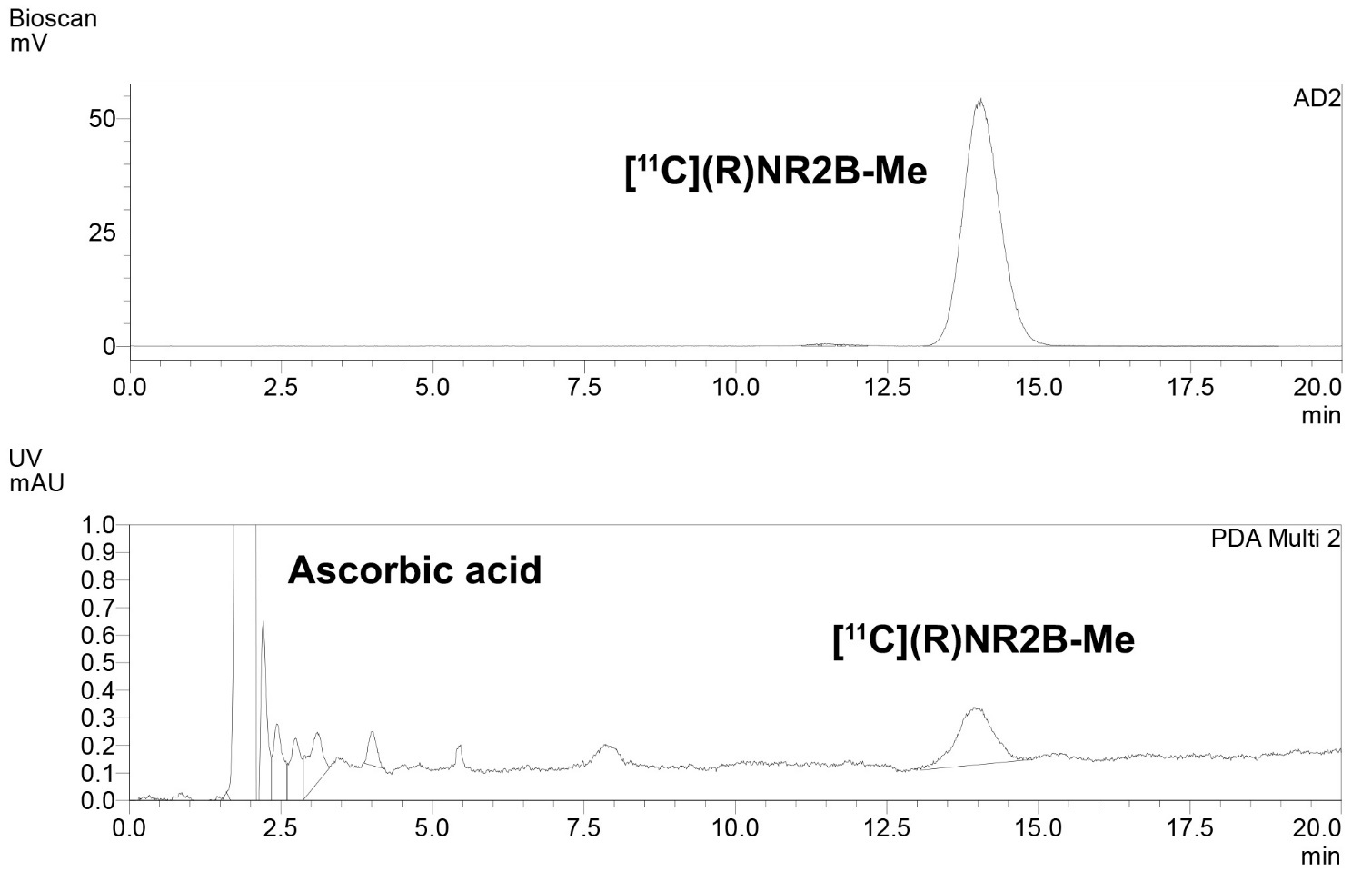


**Supplemental Figure S5**. Radiochromatogram from the radio-HPLC analysis of formulated ^11^C-(*+*)-NR2B-Me (*t*_R_ ~ 14 min). Y-axes are on linear scales. See full text for HPLC conditions and see *Radiosynthesis* and *Properties of NR2B-Me* for all details).


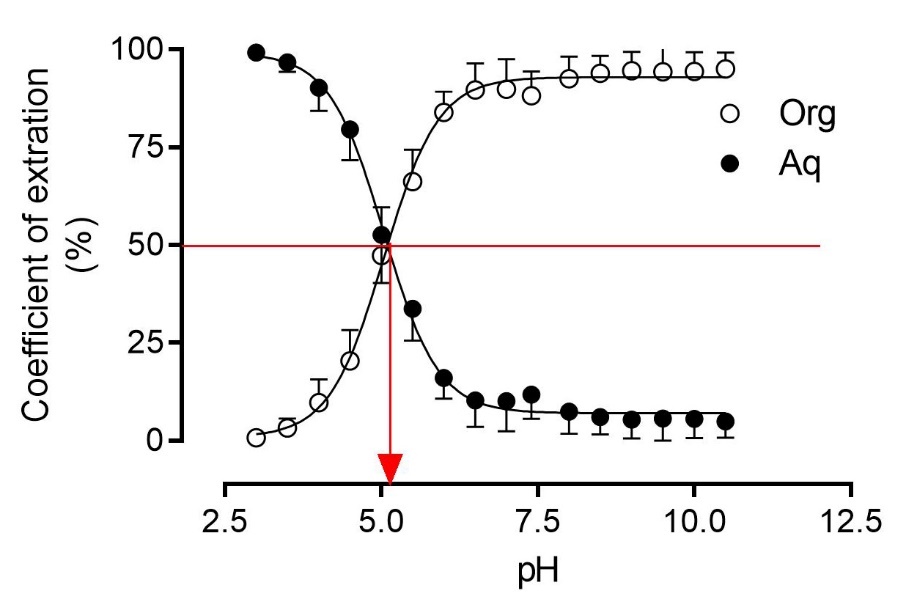


**Supplemental Figure S6**. p*H*-dependence of the distribution of ^11^C-NR2B-Me between cyclohexane and sodium phosphate buffers. Error bars are mean ± SD and are within the symbol size if not shown. The red arrow indicates the apparent p*K*a of ^11^C-NR2B-Me. The curves are fitted with Prism software (see *Measurement of LogD and pKa* and *Properties of NR2B-Me* for details).

**A**


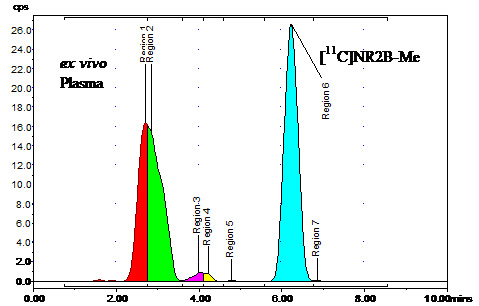


**B**


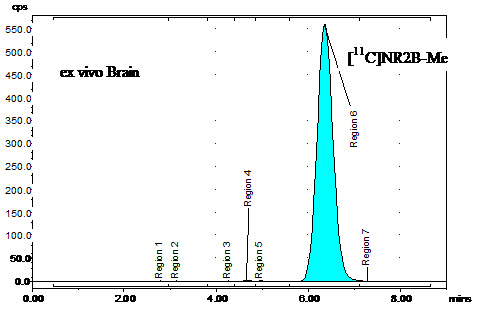


**Supplemental Figure S7**. High-performance liquid chromatography (HPLC) radiochromatograms of rat plasma (**A**) and brain (**B**) sampled 30 minutes after the intravenous injection of ^11^C-NR2B2B-Me, measured ex vivo. The radioligand represented 46.2% of the radioactivity in plasma, and 99.5% in brain. Y-axis (radioactivity; cps) is on linear scale. See full text for HPLC conditions *(*see *Radiometabolites of Racemic ^11^C-NR2B-Me in Rat Tissues Ex Vivo* for details).

**Supplementary Figure S8**. Whole rat brain TACs for ^11^C-(–)-NR2B-Me, ^11^C-(*+*)-NR2B-Me, ^11^C-(*R*)-NR2B-SMe, and ^11^C-(*S*)-NR2B-SMe (see main text, *Evaluation of* *^11^C-NR2B-Me Enantiomers in Rats using PET*).


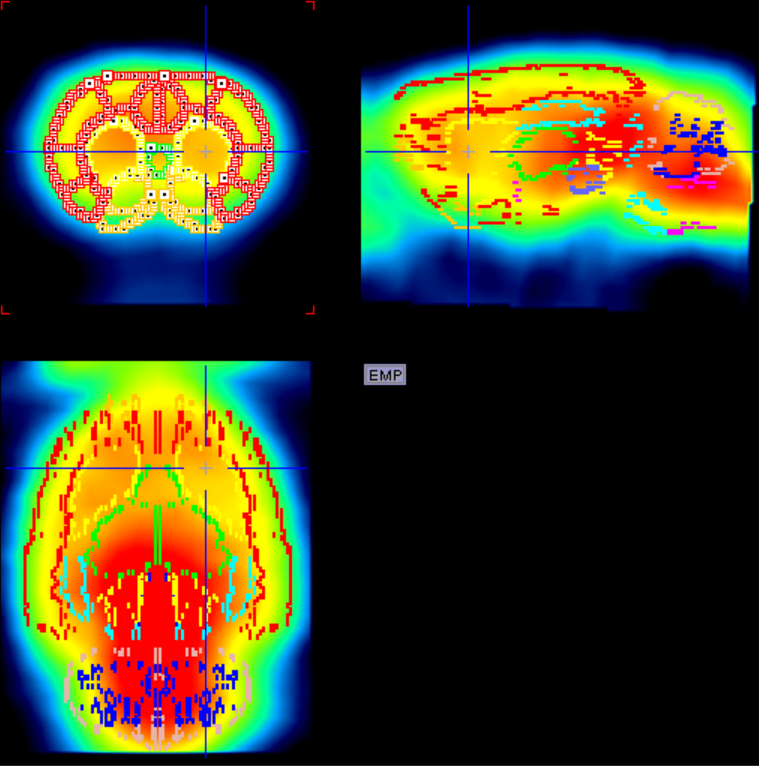


**Supplementary Figure S9**. Summed (0–90 minutes) PET image of rat brain after intravenous injection of ^11^C-(+)-NR2B-Me. Manual co-registration of different brain regions based on Schiffer’s rat template. Fourteen regions were delineated (see main text*, Evaluation of ^11^C-NR2B-Me Enantiomers in Rats using PET)*.

**Supplementary Figure S10**. Pairs of Lassen plots using standardized uptake value (SUV) for ^11^C-(–)-NR2B-Me (**A**) and ^11^C-(*+*)-NR2B-Me (**B**) in different regions of the rat brain at baseline and after pre-blocking with 0.25 mg/kg of Ro-25-6981. Estimated binding potential (*BP*_ND_) for the whole brain = SUV_BL_ / SUV_ND_ –1 ≈ 2.4/0.4 –1 = 5.0. For ^11^C-(–)-NR2B-Me (panel **A**), *BP*_ND_ for Experiment1 is between 3.4 and 5.2 and for Experiment2 between 5.0 and 7.6. For ^11^C-(+)-NR2B-Me (panel **B**), *BP*_ND_ is between 4.6 and 6.6 in Experiment1 and between 4.9 and 6.9 for Experiment2. Data are for *n* = 1 (see main text, *Evaluation of ^11^C-NR2B-Me Enantiomers in Rats using PET*).

**** ****

**Supplementary Figure S11**. *In vivo* dose-dependent pre-blocking of ^11^C-(*+*)-NR2B-Me by the NR2B ligand CO101244 (*ED*_50_ = 41 nmol/kg) in rats. Data are for *n* = 1 (see main text, *Dose Response of Candidate GluN2B Radioligands and ^18^F-FTC146 in Whole Rat Brain to GluN2B Pre-blocking Agents*).

**Supplementary Figure S12**. Pre-blocking of whole brain radioactivity uptake in rat by dosing with the σ1 ligand TC1 before intravenous injection of ^11^C-(*–*)-NR2B-Me (**A**) and ^11^C-(*+*)-NR2B-Me (**B**) and fitted dose-response curves derived from panels A and B for TC1 (**C**) (*ED*_50_ = 48 nmol/kg, and D, *ED*_50_ = 64 nmol/kg). Data are for *n* = 1 (see main text, *Dose Response of Candidate GluN2B Radioligands and ^18^F-FTC146 in Whole Rat Brain to σ1 Receptor Agonists*).

**Supplementary Figure S13**. Pre-blocking of whole brain radioactivity uptake in rat by dosing with the σ1 receptor ligand SA4503 before intravenous injection of ^11^C-(*–*)-NR2B-Me (**A**) and ^11^C-(*+*)-NR2B-Me (**B**) and fitted dose-response curves derived from panels A and B for SA4503 (**C**) (*ED*_50_ = 43 nmol/kg, and D, *ED*_50_ = 41 nmol/kg). Data are for *n* = 1 (see main text, *Dose Response of Candidate GluN2B Radioligands and ^18^F-FTC146 in Whole Rat Brain to σ1 Receptor Agonists*).

**References**

**1.** Cai L, Liow JS, Morse CL, et al. Evaluation of ^11^C-NR2B-SMe and its Enantiomers as PET Radioligands for Imaging the NR2B Subunit within the NMDA Receptor Complex in Rats. *J Nucl Med.* 2020;61:00-000.

**2.** Tewes B, Frehland B, Schepmann D, Schmidtke KU, Winckler T, Wunsch B. Design, Synthesis, and Biological Evaluation of 3-Benzazepin-1-ols as NR2B-Selective NMDA Receptor Antagonists. *ChemMedChem.* 2010;5:687-695.

**3.** Cheng Y, Prusoff WH. Relationship between Inhibition Constant (K1) and Concentration of Inhibitor Which Causes 50 Per Cent Inhibition (I50) of an Enzymatic-Reaction. *Biochemical Pharmacology.* 1973;22:3099-3108.

**4.** James ML, Shen B, Nielsen CH, et al. Evaluation of sigma-1 receptor radioligand ^18^F-FTC-146 in rats and squirrel monkeys using PET. *J Nucl Med.* 2014;55:147-153.

**5.** Briard E, Zoghbi SS, Imaizumi M, et al. Synthesis and evaluation in monkey of two sensitive ^11^C-labeled aryloxyanilide ligands for imaging brain peripheral benzodiazepine receptors in vivo. *J Med Chem.* 2008;51:17-30.

**6.** Gandelman MS, Baldwin RM, Zoghbi SS, Zea-Ponce Y, Innis RB. Evaluation of ultrafiltration for the free-fraction determination of single photon emission computed tomography (SPECT) radiotracers: ß-CIT, IBF, and iomazenil. *J Pharm Sci.* 1994;83:1014-1019.

**7.** Bradshaw TJ, Voorbach MJ, Reuter DR, Giamis AM, Mudd SR, Beaver JD. Image quality of Zr-89 PET imaging in the Siemens microPET Focus 220 preclinical scanner. *Mol Imaging Biol.* 2016;18:377-385.

**8.** Kim MJ, Shrestha SS, Cortes M, et al. Evaluation of Two Potent and Selective PET Radioligands to Image COX-1 and COX-2 in Rhesus Monkeys. *J Nucl Med.* 2018;59:1907-1912.
